# Predictors of outcomes in advanced non-small cell lung cancer treated with pembrolizumab maintenance

**DOI:** 10.1093/oncolo/oyaf070

**Published:** 2025-08-25

**Authors:** Vamsidhar Velcheti, Xuezheng Sun, Maya Hanna, Nicole M Zimmerman, Warsha K Singh, Manasee Shah, Xinmei Zhu, Anne Liao, Mehmet Altan

**Affiliations:** Division of Hematology & Oncology, Mayo Clinic Comprehensive Cancer Center, Jacksonville, FL, USA; Global Epidemiology, Oncology, GSK, Research Triangle, NC, USA; Global Epidemiology, Oncology, GSK, Collegeville, PA, USA; Real-World Biostatistics, GSK, Cleveland, OH, USA; Real-World Programming, GSK, Bengaluru, India; Global Real-World Evidence & Health Outcomes Research, Oncology, Collegeville, PA, USA; Global Real-World Evidence & Health Outcomes Research, Oncology, Collegeville, PA, USA; Global Medical Affairs, Oncology, GSK, Zug, Switzerland; Department of Thoracic/Head and Neck Medical Oncology, The University of Texas, MD Anderson Cancer Center, Houston, TX, USA

**Keywords:** non-small cell lung cancer, electronic health records, pembrolizumab, pemetrexed, retrospective studies

## Abstract

**Background:**

Real-world first-line maintenance (1LM) treatment data are limited for advanced/metastatic non-small cell lung cancer (a/mNSCLC).

**Materials and Methods:**

In this electronic health record-derived, deidentified database study, eligible patients (≥18 years; diagnosed with stage III/IV non-small cell lung cancer [June 1, 2017-September 30, 2021]) initiated pembrolizumab-based 1LM after 4-6 cycles of first-line (1L) platinum-based chemotherapy-pembrolizumab ± pemetrexed. Study outcomes were real-world time to next treatment or death (rwTTNTD), overall survival (rwOS), and predictors of outcomes.

**Results:**

Of 1944 patients analyzed (median follow-up, 12.2 months), 51.9% received 1LM pembrolizumab-pemetrexed and 48.1% pembrolizumab monotherapy. Median rwTTNTD and rwOS were 9.2 (95% CI: 8.5-9.8) and 18.7 (95% CI: 17.7-20.3) months, respectively. In multivariable analyses, factors significantly associated with shorter rwTTNTD included 5%-<10% (hazard ratio [HR], 1.34; 95% CI: 1.17-1.54) or ≥10% (HR, 1.69; 95% CI: 1.42-2.01) weight loss during 1L versus 0% or <5% weight loss. Programmed death-ligand 1 (PD-L1) expression 1%–49% (HR, 0.81; 95% CI: 0.71-0.93) or ≥50% (HR, 0.55; 95% CI: 0.47-0.64) and female sex (HR, 0.85; 95% CI: 0.75-0.95) were significantly associated with longer rwTTNTD. These variables were also significantly associated with shorter (weight loss 5%-<10%: HR, 1.52; 95% CI: 1.30-1.77; ≥10% HR, 2.06; 95% CI: 1.71-2.48) and longer rwOS (PD-L1 expression: 1%-49% HR, 0.84; 95% CI: 0.72-0.98; ≥ 50% HR, 0.57; 95% CI: 0.48-0.68; female sex: HR, 0.81; 95% CI: 0.71-0.92).

**Conclusions:**

Predictors of real-world clinical outcomes included 1L treatment, weight loss, PD-L1 status, and sex. Poor outcomes persisted despite immunotherapy-based 1LM availability, revealing an unmet need in this population.

**Implications for Practice**

It is unknown which characteristics are predictive of real-world outcomes in patients with advanced/metastatic non-small cell lung cancer (a/mNSCLC) receiving pembrolizumab-based first-line maintenance (1LM). In this real-world study of 1944 patients with a/mNSCLC receiving pembrolizumab-based 1LM, weight loss during first-line treatment was associated with significantly shorter real-world overall survival (rwOS) and time to next treatment or death (rwTTNTD). Higher programmed death-ligand 1 percentage and female sex were associated with significantly longer rwOS and rwTTNTD. These findings may help physicians identify patients with a/mNSCLC who respond better to platinum-based first-line induction chemotherapy with pembrolizumab, followed by pembrolizumab-based 1LM therapy.

## Introduction

Lung cancer represents the leading cause of cancer-related deaths, both in the United States and globally.^1,2^ Non-small cell lung cancer (NSCLC) accounts for 80%-85% of lung cancer cases, of which 25%-30% are squamous cell carcinomas and 70%-75% are nonsquamous carcinomas.^3^ Most patients with NSCLC present with advanced disease at diagnosis (with stage IIIB accounting for ≈14% and stage IV accounting for up to 48%).^4,5^ Despite treatment advances, outcomes for NSCLC remain poor, particularly among patients with advanced/metastatic (a/m) disease, with 5-year relative survival rates decreasing dramatically as disease stage increases (stage IIIa: 26.2%; stage IIIb: 17.3%; and stage IV: 5.8%).^5^

The preferred first-line (1L) treatment for a/mNSCLC without actionable mutations has long consisted of platinum-based, 2-drug combination chemotherapy regimens^6^; however, the introduction of immunotherapy has provided alternative options,^7^ and treatment with immune checkpoint inhibitors targeting programmed cell death protein 1 (PD-1) or programmed death-ligand 1 (PD-L1) is common. In the KEYNOTE-407 study of patients with squamous a/mNSCLC, the PD-1 inhibitor pembrolizumab in combination with carboplatin plus paclitaxel or paclitaxel (protein-bound) significantly prolonged progression-free survival (PFS) and overall survival (OS) relative to chemotherapy alone.^8^ In the KEYNOTE-189 study of patients with nonsquamous a/mNSCLC, those receiving pemetrexed plus platinum-based chemotherapy followed by pembrolizumab-based maintenance demonstrated prolonged PFS and OS compared with those not receiving pembrolizumab-based maintenance therapy.^9-11^ However, real-world data on treatment patterns and clinical outcomes, particularly in the maintenance setting, are limited.^12^

The objectives of this study were to describe treatment patterns and clinical outcomes and identify factors associated with clinical outcomes in patients with a/mNSCLC receiving pembrolizumab-based first-line maintenance (1LM) therapy in the United States.

## Materials and methods

### Study design and patient population

This US-based retrospective observational study used the nationwide Flatiron Health electronic health record-derived deidentified database (June 1, 2017-March 31, 2022), with patient-level structured and unstructured data, curated via technology-enabled abstraction from approximately 280 cancer clinics, representing an estimated 800 sites of care across the United States.^13,14^ The deidentified data were subject to obligations to prevent reidentification and protect patient confidentiality. Patients diagnosed with lung cancer per International Classification of Disease (ICD) codes (ICD-9: 162x; ICD-10: C34x, C39.9) during the patient selection period (June 1, 2017, to September 30, 2021) were eligible if they were aged ≥18 years at the index date; had stage IIIB, IIIC, or IV NSCLC at initial diagnosis; and initiated pembrolizumab-based 1LM therapy (the date of first administration was defined as the index date) after completing 4-6 cycles of 1L platinum-based induction chemotherapy in combination with pembrolizumab ± pemetrexed. First-line induction and 1LM therapy with pembrolizumab were defined using a rule-based, oncology clinician-defined algorithm. Patients with known *ALK*, *EGFR*, *ROS1*, or *BRAF* genomic aberrations, who lacked structured activity in the 90 days post-diagnosis, and who received 1L treatment with a poly(ADP-ribose) polymerase (PARP) inhibitor before the index date were excluded. Patients were followed from the index date until the end of the study (March 31, 2022), loss to follow-up, or death, whichever occurred first.

### Study outcomes

Baseline patient demographics, clinical characteristics, and pembrolizumab-based 1LM treatment characteristics were collected. Weight loss was defined as the recorded weight on the date nearest to the initial diagnosis minus the recorded weight on the date nearest to the index, with time points separated by ≥2 months. Outcomes of interest included median duration of 1L treatment, real-world time to next treatment or death (rwTTNTD), and real-world OS (rwOS). Duration of maintenance therapy was measured from the first recorded drug episode date to the last recorded drug episode date within the 1LM setting. Patients were followed to the earliest of last structured activity or end of study period if they did not receive second-line (2L) therapy, die, or have a structured visit date >120 days after the last drug episode date in the 1LM setting. rwTTNTD was measured from the index date to the start of 2L therapy or the date of death, whichever occurred first. rwOS was measured from the index date until the date of death. rwTTNTD and rwOS were censored at the end of the study period or the date of the last known activity (whichever occurred first). Outcomes were examined in the overall cohort and stratified by tumor histology (nonsquamous versus squamous). In patients with nonsquamous histology, outcomes were further stratified by receipt of pemetrexed during 1L therapy (yes or no).

### Data analysis

Descriptive statistics were used to summarize baseline demographics, clinical characteristics, and pembrolizumab-based 1LM treatment patterns and outcomes. To identify clinical and demographic predictors of rwTTNTD and rwOS, univariable Cox proportional hazards models and log-rank tests were conducted to assess the association between each risk factor and each outcome in the overall cohort and by subgroups (histology, stage, and receipt of 1L pemetrexed [yes/no]). Multivariable Cox proportional hazards regression models were fit to estimate the adjusted associations between risk factors and outcomes. Models included demographic and clinicopathological factors, comorbidities, prior treatment (Table S1), and factors associated with each outcome in the univariable analysis with a log-rank *P < *.10. The proportional hazard hypothesis was verified with the Schoenfeld residual method. All tests were 2-sided with a significance criterion of .05. A sensitivity analysis using machine-learning approaches was conducted to assess the consistency of the primary analysis results for rwOS outcomes (Supplementary Methods).

## Results

### Patient demographics and clinical characteristics

**Figure 1.**
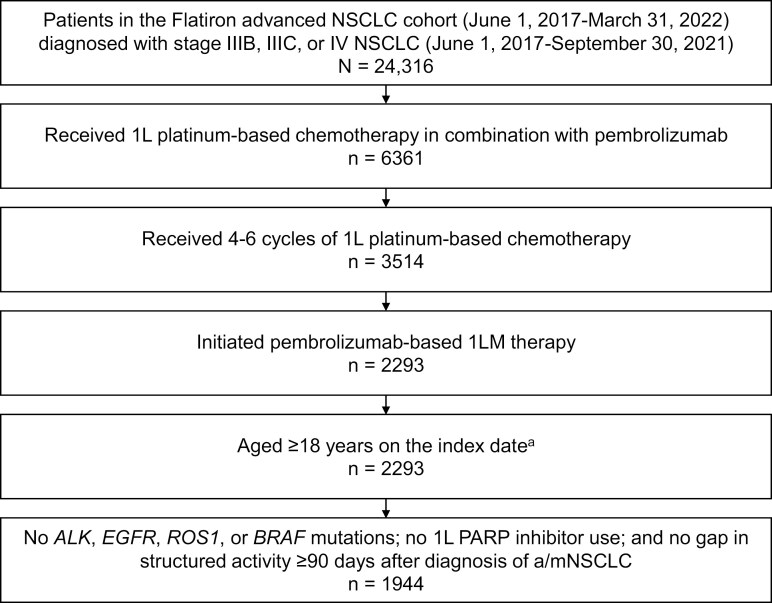
Study population. Abbreviations: 1L, first-line; 1LM, first-line maintenance; a/m, advanced/metastatic; NSCLC, non-small cell lung cancer; PARP, poly(ADP-ribose) polymerase. ^a^Defined as the date on which pembrolizumab-based 1LM therapy was first administered.

Of the 24 316 patients from the database diagnosed with a/mNSCLC during the study period, 1944 satisfied all eligibility criteria and were included in this study (Figure 1). Most patients were aged 65-79 years (53.8%), were White (68.1%), had stage IV NSCLC at diagnosis (96.8%), had an Eastern Cooperative Oncology Group (ECOG) performance status score of 0-1 at the time of 1L treatment initiation (74.6%), and had received 4 cycles of 1L platinum-based chemotherapy (80.0%; Table 1). Patients had PD-L1 expression levels of <1% (29.5%), 1%-49% (29.2%), ≥50% (23.0%), and unknown status/not tested (18.3%). Most patients (77.1%) had nonsquamous histology. Demographic and clinical characteristics were generally similar between the 2 histologic subgroups.

**Table 1.** Demographic, clinical, and treatment characteristics of patients who initiated 1LM therapy (overall and by tumor histology).

		Tumor histology	
Characteristic	Patients (*N* = 1944)	Nonsquamous (*n* = 1498)	Squamous (*n* = 359)
Age, y			
Mean (SD)	67 (9)	67 (9)	69 (8)
Median (IQR)	68 (61-74)	68 (61-74)	70 (63-75)
Age group, y			
18-64	715 (36.8)	572 (38.2)	103 (28.7)
65-79	1046 (53.8)	790 (52.7)	217 (60.4)
≥80	183 (9.4)	136 (9.1)	39 (10.9)
Sex			
Male	1057 (54.4)	765 (51.1)	236 (65.7)
Female	887 (45.6)	733 (48.9)	123 (34.3)
Race			
White	1323 (68.1)	996 (66.5)	265 (73.8)
Black/African American	205 (10.5)	171 (11.4)	26 (7.2)
Asian	18 (0.9)	18 (1.2)	0 (0.0)
Other	200 (10.3)	159 (10.6)	32 (8.9)
Unknown/missing	198 (10.2)	154 (10.3)	36 (10.0)
Ethnicity			
Not Hispanic or Latino	1360 (70.0)	1051 (70.2)	246 (68.5)
Hispanic or Latino	63 (3.2)	49 (3.3)	13 (3.6)
Unknown/missing	521 (26.8)	398 (26.6)	100 (27.9)
Region of residence			
South	866 (44.5)	653 (43.6)	172 (47.9)
Northeast	354 (18.2)	279 (18.6)	59 (16.4)
Midwest	271 (13.9)	205 (13.7)	55 (15.3)
West	239 (12.3)	192 (12.8)	38 (10.6)
Other/unknown	214 (11.0)	169 (11.3)	35 (9.7)
Practice type^a^			
Community	1763 (90.7)	1355 (90.5)	331 (92.2)
Academic^b^	191 (9.8)	149 (9.9)	32 (8.9)
Histology			
Nonsquamous	1498 (77.1)	1498 (100.0)	0 (0.0)
Squamous	359 (18.5)	0 (0.0)	359 (100.0)
Not otherwise specified	87 (4.5)	—	—
History of smoking			
Yes	1768 (90.9)	1339 (89.4)	345 (96.1)
No	176 (9.1)	159 (10.6)	14 (3.9)
Disease stage at diagnosis			
III	63 (3.2)	39 (2.6)	20 (5.6)
IV	1881 (96.8)	1459 (97.4)	339 (94.4)
ECOG PS score at initial diagnosis			
0-1	1451 (74.6)	1122 (74.9)	266 (74.1)
>1	241 (12.4)	173 (11.5)	55 (15.3)
Unknown	252 (13.0)	203 (13.6)	38 (10.6)
PD-L1 status			
<1%	573 (29.5)	437 (29.2)	113 (31.5)
1%-49%	568 (29.2)	439 (29.3)	111 (30.9)
≥50%	447 (23.0)	354 (23.6)	69 (19.2)
Unknown/not tested	356 (18.3)	268 (17.9)	66 (18.4)
1L induction treatment regimen^c^			
Platinum-based chemotherapy + pembrolizumab + pemetrexed	1503 (77.3)	1435 (95.8)	5 (1.4)
Platinum-based chemotherapy + pembrolizumab	418 (21.5)	47 (3.1)	351 (97.8)
Other	23 (1.2)	16 (1.1)	3 (0.8)
No. of 1L platinum-based chemotherapy cycles received			
4	1555 (80.0)	1187 (79.2)	301 (83.8)
5	127 (6.5)	107 (7.1)	17 (4.7)
6	262 (13.5)	204 (13.6)	41 (11.4)
Duration of 1L therapy, mean (SD), mo			
Overall	2.5 (0.7)	2.5 (0.7)	2.7 (0.8)
1LM regimens			
Pembrolizumab + pemetrexed	1009 (51.9)	965 (64.4)	2 (0.6)
Pembrolizumab	935 (48.1)	533 (35.6)	357 (99.4)
Duration of 1LM therapy, median (IQR), mo			
Overall	6.2 (2.5-18.2)	6.2 (2.6-17.7)	5.5 (2.1-20.2)
Regimen			
Pembrolizumab + pemetrexed	6.9 (3.0-19.3)	7.0 (3.0-19.1)	2.1 (2.1-2.1)
Pembrolizumab alone	5.5 (2.1-17.3)	5.1 (2.1-15.2)	5.5 (2.1-20.20)

Values shown are *n* (%) unless otherwise stated.

Abbreviations: 1L, first-line; 1LM, first-line maintenance; ECOG PS, Eastern Cooperative Oncology Group performance status; PD-L1, programmed death-ligand 1.

^a^Patients with records in academic and community practices are counted in both categories; therefore, patient counts may sum to more than 100%.

^b^Academic may include both university and nonuniversity academic settings.

^c^A rules-based, oncologist-defined approach was used to define lines of therapy.

### Treatment patterns in the 1L setting

**Figure 2.**
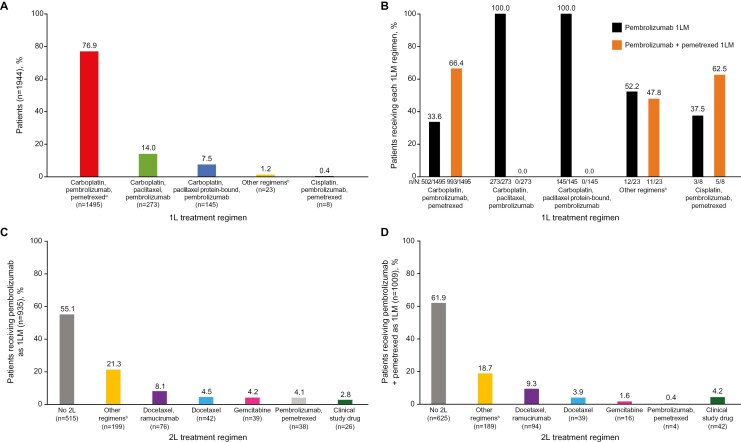
Treatment in the overall cohort. The 1L treatment regimen for patients in the overall cohort (*n* = 1944) (**A**), the proportion of patients who received pembrolizumab or pembrolizumab + pemetrexed as 1LM based on 1L treatment regimen (**B**), the 2L treatment regimen for patients who received pembrolizumab as 1LM (*n* = 935) (**C**), and 2L treatment regimen for patients who received pembrolizumab + pemetrexed as 1LM (*n* = 1009) (**D**). Abbreviations: 1L, first-line; 1LM, first-line maintenance; 2L, second-line. ^a^This group includes 1468 patients (75.5%) treated with carboplatin, pembrolizumab, and pemetrexed, and 27 patients (1.4%) treated with abiraterone, carboplatin, pembrolizumab, and pemetrexed. ^b^Other regimens include regimens not defined in previous drug classes, such as sotorasib, capmatinib, and gemcitabine/vinorelbine.

Roughly equal proportions of patients received 1LM pembrolizumab plus pemetrexed (51.9% [1009/1944]) or pembrolizumab alone (48.1% [935/1944]). Two-thirds of patients who received 1L induction therapy with carboplatin, pembrolizumab, and pemetrexed were prescribed 1LM pembrolizumab plus pemetrexed (Figure 2B). All patients who received carboplatin, paclitaxel, and pembrolizumab or carboplatin, paclitaxel (protein-bound), and pembrolizumab as 1L induction therapy received 1LM pembrolizumab monotherapy (Figure 2B). In the overall study population, 2L treatment was not observed for >50% of patients (Figure 2C and D).

Most patients with nonsquamous histology received carboplatin, pembrolizumab, and pemetrexed as 1L induction therapy (95.4% [1428/1498]; Figure S1A). Patients with nonsquamous histology primarily received 1LM pembrolizumab plus pemetrexed (64.4% [965/1498]), although 35.6% (533/1498) received 1LM pembrolizumab monotherapy (Table 1). Most patients with squamous histology received either carboplatin, paclitaxel, and pembrolizumab (62.7% [225/359]) or carboplatin, paclitaxel (protein-bound), and pembrolizumab (34.5% [124/359]) as 1L induction therapy and 99.4% (357/359) of patients with squamous histology received 1LM pembrolizumab monotherapy (Table 1 and Figure S2A).

### 1LM treatment duration

The median duration of 1LM was 6.2 months for the overall population, 6.2 months for patients with nonsquamous histology, and 5.5 months for patients with squamous histology (Table 1). In the overall population, the median duration of 1LM was 6.9 months for patients receiving pembrolizumab plus pemetrexed and 5.5 months for those receiving pembrolizumab monotherapy (Table 1). The median duration of 1LM pembrolizumab monotherapy was 5.1 months for patients with nonsquamous histology and 5.5 months for patients with squamous histology (Table 1). In subgroups defined by baseline characteristics, the median duration of pembrolizumab-based 1LM was shorter for patients with an ECOG performance score of > 1 (4.4 months) than for patients with a score of 0-1 (6.4 months) and longer for patients with a PD-L1 status of ≥50% (8.6 months) than for patients with a PD-L1 status of <1% (4.8 months) or 1%-49% (6.2 months).

### Unadjusted rwTTNTD and rwOS

**Figure 3.**
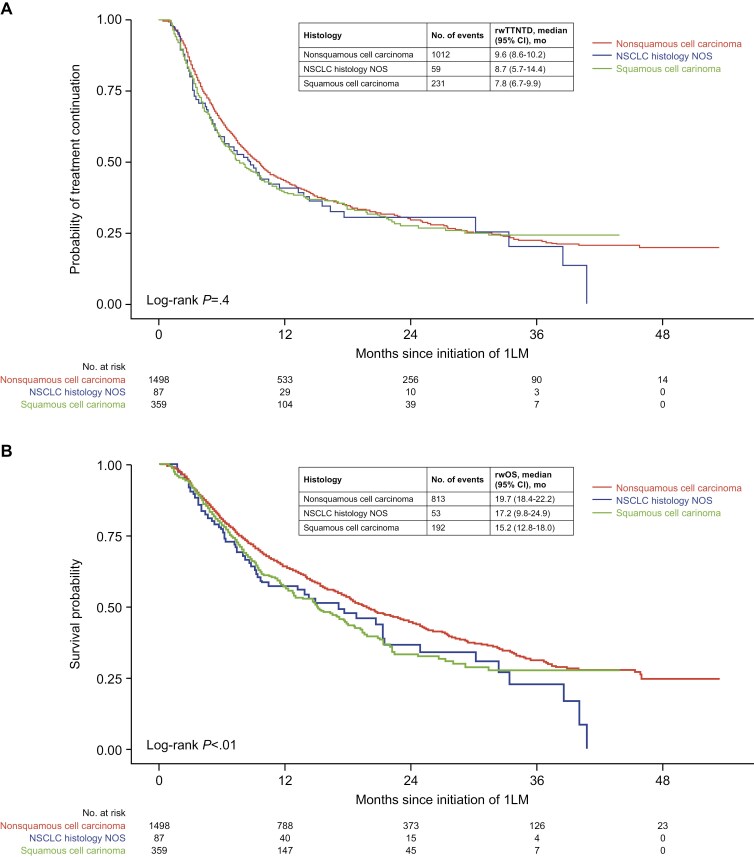
(**A**) rwTTNTD and (**B**) rwOS by histology. Abbreviations: 1LM, first-line maintenance; NOS, not otherwise specified; NSCLC, non-small cell lung cancer; rwOS, real-world overall survival; rwTTNTD, real-world time to next treatment or death.

During a median follow-up of 12.2 months, 1302 patients advanced to the next treatment or died. In the overall cohort, 1058 patients died. Median rwTTNTD was 9.2 months (95% CI: 8.5-9.8), and median rwOS was 18.7 (95% CI: 17.7-20.3) months. rwTTNTD did not differ significantly for patients with nonsquamous versus squamous histology (9.6 vs. 7.8 months; *P* = .2; Figure 3A); however, rwOS was significantly longer for patients with nonsquamous than for those with squamous histology (19.8 vs. 15.2 months; *P* = .006; Figure 3B). While rwTTNTD differed significantly for patients with nonsquamous histology who received 1L induction pemetrexed versus those who did not (9.7 vs. 5.9 months; *P* = .04; Figure S3A), rwOS did not (20.0 vs. 17.7 months; *P* = .5; Figure S3B). Given the small proportion of patients with nonsquamous histology who did not receive 1L pemetrexed (4.2% [63/1498]), these results should be interpreted with caution. Neither rwTTNTD, nor rwOS differed significantly for patients with stage III disease versus those with stage IV disease (Figure S4).

In unadjusted analyses, factors significantly associated with longer rwTTNTD were female sex (hazard ratio [HR], 0.82; 95% CI: 0.73-0.91; *P* < .01) and higher PD-L1 expression (0%-<1% vs. ≥50%: HR, 0.57; 95% CI: 0.49-0.66; *P* < .0001; 0%-<1% vs. 1%-49%, HR, 0.82; 95% CI: 0.71-0.94; *P* < .01). Factors significantly associated with longer rwOS were female sex (HR, 0.76; 95% CI: 0.68-0.86; *P* < .0001) and higher PD-L1 expression (0%-<1% vs. ≥50%: HR, 0.57; 95% CI: 0.48-0.68; *P* < .0001; 0%-<1% vs. 1%-49%, HR, 0.84; 95% CI: 0.72-0.98; *P = *.02).

### Predictors of rwTTNTD and rwOS

**Figure 4.**
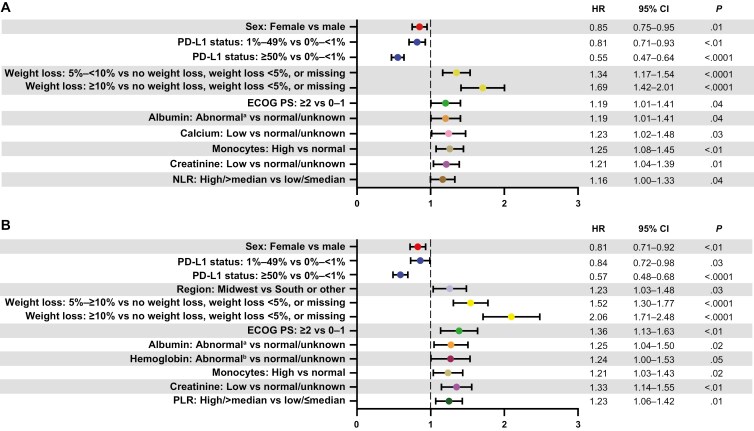
Predictors of (**A**) rwTTNTD and (**B**) rwOS in multivariable analyses. For rwTTNTD (**A**), index year (2017, 2019, 2020, and 2021 vs. 2018), index age (65-79 years and ≥ 80 years vs. 18-64 years), ethnicity (Hispanic/Latino vs not Hispanic/Latino), region (Northeast, Midwest, West, and unknown vs. South or other), histology (NSCLC NOS and squamous cell carcinoma vs. nonsquamous cell carcinoma), smoking history (history of smoking vs. no history of smoking), hemoglobin (abnormal^b^ vs. normal/unknown), aspartate aminotransferase (abnormal vs. normal/unknown), calcium (high vs. normal/unknown), monocytes (low and unknown vs. normal), alanine aminotransferase (low and high vs. normal/unknown), creatinine (high vs. normal/unknown), and NLR (unknown vs. low/≤median) were also evaluated and shown to be nonsignificant. For rwOS (**B**), index year (2017, 2019, 2020, and 2021 vs. 2018), index age (65-79 years and ≥80 years vs. 18-64 years), region (Northeast, West, and unknown vs. South or other), ECOG PS (unknown vs. 0-1), histology (NSCLC NOS and squamous cell carcinoma vs. nonsquamous cell carcinoma), cardiovascular disease (yes vs. no), calcium (low and high vs. normal/unknown), monocytes (low and unknown vs. normal), alanine aminotransferase (low and high vs. normal/unknown), creatinine (high vs. normal/unknown), NLR (high/>median vs. low/≤median), and PLR (unknown vs. low/≤median) were also evaluated and shown to be nonsignificant. Abbreviations: ECOG PS, Eastern Cooperative Oncology Group performance status; HR, hazard ratio; NLR, neutrophil-to-lymphocyte ratio; NOS, not otherwise specified; NSCLC, non-small cell lung cancer; PD-L1, programmed death-ligand 1; PLR, platelet-to-lymphocyte ratio; rwOS, real-world overall survival; rwTTNTD, real-world time to next treatment or death. ^a^Consists of clinically low albumin levels (*n* = 262) versus high albumin levels (*n* = 1). ^b^Consists of clinically low hemoglobin levels (*n* = 1721) versus high hemoglobin levels (*n* = 2).

In multivariable analyses in the overall population, factors significantly associated with longer rwTTNTD included female sex (HR, 0.85; 95% CI: 0.75-0.95) and higher PD-L1 expression (0%-<1% vs. ≥50%: HR, 0.55; 95% CI: 0.47-0.64; 0%-<1% vs. 1%-49%, HR, 0.81; 95% CI: 0.71-0.93). Factors significantly associated with shorter rwTTNTD included greater weight loss during 1L treatment (≥10% vs. 0% or <5%: HR, 1.69; 95% CI: 1.42-2.01; 5%-10% vs. 0% or <5%: HR, 1.34; 95% CI: 1.17-1.54), higher ECOG performance status scores (≥2 vs. 0-1: HR, 1.19; 95% CI: 1.01-1.41), abnormal (low or high) albumin levels (vs. normal/unknown: HR, 1.19; 95% CI: 1.01-1.41), low calcium level (vs. normal/unknown: HR, 1.23; 95% CI: 1.02-1.48), high monocyte count (vs. normal: HR, 1.25; 95% CI: 1.08-1.45), low creatinine value (vs. normal/unknown: HR, 1.21; 95% CI: 1.04-1.39), and high/greater than the median (vs. low: HR, 1.16; 95% CI: 1.00-1.33) neutrophil-to-lymphocyte ratio (Figure 4A).

Factors significantly associated with longer rwOS included female sex (HR, 0.81; 95% CI: 0.71-0.92) and higher PD-L1 expression (0%-<1% vs. ≥50%: HR, 0.57; 95% CI: 0.48-0.68; 0%-<1% vs. 1%-49%: HR, 0.84; 95% CI: 0.72-0.98). Factors significantly associated with shorter rwOS included greater weight loss during 1L treatment (≥10% vs. 0% or <5%: HR, 2.06; 95% CI: 1.71-2.48; 5%-10% vs. 0% or <5%: HR, 1.52; 95% CI: 1.30-1.77), living in the Midwest (vs. the South or other US regions: HR, 1.23; 95% CI: 1.03-1.48), higher ECOG performance status scores (≥2 vs. 0-1: HR, 1.36; 95% CI: 1.13-1.63), abnormal (low or high) albumin levels (vs. normal/unknown: HR, 1.25; 95% CI: 1.04-1.50), abnormal (low or high) hemoglobin levels (vs. normal/unknown: HR, 1.24; 95% CI: 1.00-1.53), high monocyte count (vs. normal: HR, 1.21; 95% CI: 1.03-1.43), low creatinine levels (vs. normal/unknown: HR, 1.33; 95% CI: 1.14-1.55), and high/greater-than-the-median (vs. low: HR, 1.23; 95% CI: 1.06-1.42) platelet-to-lymphocyte ratio (Figure 4B). Similar factors were predictive of rwTTNTD and rwOS in patients with squamous (Tables S2 and S3) and nonsquamous (Tables S4 and S5) histology. However, fewer significant associations were identified among patients with squamous histology for rwTTNTD, potentially because the small sample size limited statistical power.

In the sensitivity analysis, machine-learning models supported the general trends observed in the original Cox proportional hazards analysis while accounting for limitations intrinsic to Cox modeling (Tables S6 and S7).

## Discussion

This retrospective electronic health record-derived database study described patient and disease characteristics, 1L treatment duration, clinical outcomes (rwTTNTD and rwOS), and outcome predictors among patients in the United States with a/m NSCLC who completed 1L platinum-based induction chemotherapy with pembrolizumab and received pembrolizumab-based 1LM. Like other real-world studies, most patients were White, had a history of smoking, had stage IV disease at diagnosis, had nonsquamous histology, and were aged ≥65 years.^5,12,15,16^

In this study, the median duration of 1LM was 6.9 months for patients receiving pembrolizumab plus pemetrexed and 5.5 months for those receiving pembrolizumab monotherapy. These treatment durations were longer than those reported in a recent retrospective observational study by Aggarwal et al. of patients with nonsquamous a/mNSCLC treated with 1L pemetrexed, pembrolizumab, and platinum-based chemotherapy followed by pembrolizumab ± pemetrexed 1LM (median treatment duration in patients receiving pembrolizumab plus pemetrexed 1LM, 2.8 months; median treatment duration in patients receiving pembrolizumab monotherapy 1LM, 3.5 months).^12^ This difference may have been due, at least in part, to differences in the study populations. For example, this study included patients with squamous and nonsquamous a/mNSCLC and excluded those with actionable mutations, while the Aggarwal et al. study excluded patients with squamous a/mNSCLC and included those with *EGFR*, *ALK*, *KRAS*, *ROS1*, and *BRAF* mutations.^12^ Furthermore, our study had a higher proportion of patients with an ECOG performance status of 0 or 1 than the Aggarwal et al. study (74.6% vs. 61.3%).^12^

Median rwTTNTD (9.6 months) and median rwOS (19.7 months) in patients with nonsquamous a/mNSCLC in this study were similar to the median PFS (9.0 months) and median OS (22.0 months) observed in patients with nonsquamous a/mNSCLC receiving pembrolizumab plus pemetrexed 1LM in KEYNOTE-189.^10^ Median rwOS in our study was also consistent with the reported unadjusted median OS in patients with nonsquamous a/mNSCLC receiving pembrolizumab ± pemetrexed 1LM in the Aggarwal et al. study (21.0 months),^12^ suggesting that the benefits of pembrolizumab-based 1LM observed in KEYNOTE-189 study extend to real-world community-based practice.

In the multivariable analysis in the overall cohort, weight loss during 1L treatment was a key driver of shorter rwOS and rwTTNTD outcomes. This observation is supported by another medical records-based study of patients with NSCLC, which showed that increasing weight loss during the 5 months after baseline and lower body mass index at baseline were associated with substantially worse outcomes, independent of other variables.^17^ In contrast, female sex and high PD-L1 status were associated with longer rwOS and rwTTNTD. These findings are supported by one meta-analysis that demonstrated a 27% reduced risk of death in women compared with men,^18^ as well as the observation from another meta-analysis that high PD-L1 levels improved treatment outcomes in patients receiving anti–PD-1/anti–PD-L1 agents (including pembrolizumab).^19^

The results of this study should be considered within the context of several potential limitations. Our data set may not reflect the entire US population of patients with a/mNSCLC, as data were collected primarily from community-based practices; thus, our findings may not fully capture treatment practices at major university or academic centers. In addition, the data set was limited to information that was recorded in, and subsequently abstracted from, patients’ electronic health records. Information regarding reasons for treatment discontinuation and certain tumor-specific characteristics (including PD-L1 levels, which were unavailable for approximately 18% of patients in this study) were incomplete. Potential misclassification of 1LM as 2L therapy may have occurred because treatment gaps exceeding 120 days did not advance the line of therapy according to the established line-of-therapy algorithm (although this only applied to ≈4% of patients in this study). Additionally, real-world PFS data were unavailable, so rwTTNTD was used as a surrogate. As previously reported, rwTTNTD may conflate toxicity and progression as reasons for treatment change and is negated for patients who do not receive subsequent treatment post-progression.^20^ Lastly, because this study took place during the COVID-19 pandemic, we cannot exclude the possibility that patients may have missed visits or healthcare providers may have changed practices to enable remote/telemedicine visits, which may have impacted data collection and treatment. However, this is unlikely, as sensitivity analyses of rwTTNTD and rwOS for the pre-COVID-19 subgroup identified similar predictive risk factors to those identified in the overall cohort (data not shown).

Median rwOS and rwTTNTD in this real-world population were less than 2 years, highlighting the need to identify therapeutic regimens that further prolong survival. The ability of combination strategies with monoclonal antibodies targeting vascular endothelial growth factor receptor 2 or coinhibitory molecules (eg, TIM-3 and TIGIT) and other pharmacologic approaches that may synergize with anti-PD-1/PD-L1 therapies (eg, PARP inhibitors) to increase responses to front-line therapies and delay acquired resistance to immunotherapy is being investigated.^21-25^ Preclinical data demonstrating crosstalk between PARP inhibition and tumor-associated immunosuppression in vivo^26^ have prompted the clinical evaluation of combination therapy with PARP inhibitors and immunotherapy. In the phase 2 JASPER (NCT03308942) trial, combination therapy with the PARP inhibitor niraparib and pembrolizumab in patients with a/mNSCLC resulted in an objective response rate of 56.3%, with 2 complete responses and 7 partial responses; median PFS was 8.4 months.^27^ The efficacy and safety of niraparib plus pembrolizumab versus placebo plus pembrolizumab as 1LM therapy are being evaluated in patients with a/mNSCLC without actionable mutations in the phase 3 ZEAL-1L (NCT04475939) study.^28^ Additional ongoing PARP inhibitor and immunotherapy combination trials in patients with a/mNSCLC include the phase 2 ORION (NCT03775486) study of 1LM olaparib plus durvalumab versus durvalumab monotherapy^29^ and the phase 3 KEYLYNK-006 (NCT03976323) study of 1LM olaparib plus pembrolizumab versus pemetrexed plus pembrolizumab.^30^ The phase 3 KEYLYNK-008 (NCT03976362) study of 1LM olaparib plus pembrolizumab versus pembrolizumab plus placebo was terminated early based on the results of an interim analysis that failed to demonstrate an OS improvement.^31,32^ The results of our study may help guide the design of future trials by identifying patients with a/mNSCLC who stand to gain the most benefit from pembrolizumab-based 1LM regimens.

## Conclusion

The real-world treatment patterns observed in this study may improve our understanding of 1LM therapy for patients with a/mNSCLC who received 1L platinum-based induction chemotherapy with pembrolizumab in the US community-based practice setting. The short rwOS and rwTTNTD reinforce the need to identify therapeutic regimens that improve clinical outcomes. The primary risk factor driving poor outcomes with current 1LM treatment, identified for both rwOS and rwTTNTD, was greater weight loss during 1L treatment. The main predictors of improved outcomes, identified for both rwOS and rwTTNTD, were PD-L1 status of ≥50% and female sex. These findings may help physicians identify patients who respond better to 1L platinum-based induction chemotherapy with pembrolizumab, followed by pembrolizumab-based 1LM.

## Supplementary Material

oyaf070_suppl_Supplementary_Tables_S1-S7_Figures_S1-S4

## Data Availability

The data that support the findings of this study were originated by and are the property of Flatiron Health, Inc., which has restrictions prohibiting the authors from making the data set publicly available. Requests for data sharing by license or by permission for the specific purpose of replicating results in this manuscript can be submitted to PublicationsDataAccess@flatiron.com.

## References

[1] Siegel RL , Miller KD, Wagle NS, Jemal A. cancer statistics, 2023. CA Cancer J Clin. 2023;73:17-48. https://doi.org/10.3322/caac.21763 36633525

[2] Sung H , Ferlay J, Siegel RL, et al Global cancer statistics 2020: GLOBOCAN estimates of incidence and mortality worldwide for 36 cancers in 185 countries. CA Cancer J Clin. 2021;71:209-249. https://doi.org/10.3322/caac.21660 33538338

[3] Schabath MB , Cote ML. Cancer progress and priorities: lung cancer. Cancer Epidemiol Biomarkers Prev. 2019;28:1563-1579. https://doi.org/10.1158/1055-9965.EPI-19-0221 31575553 PMC6777859

[4] Majeed U , Manochakian R, Zhao Y, Lou Y. Targeted therapy in advanced non-small cell lung cancer: current advances and future trends. J Hematol Oncol. 2021;14:108. https://doi.org/10.1186/s13045-021-01121-2 34238332 PMC8264982

[5] Ganti AK , Klein AB, Cotarla I, Seal B, Chou E. Update of incidence, prevalence, survival, and initial treatment in patients with non-small cell lung cancer in the US. JAMA Oncol. 2021;7:1824-1832. https://doi.org/10.1001/jamaoncol.2021.4932 34673888 PMC8532041

[6] Wang C , Kulkarni P, Salgia R. Combined checkpoint inhibition and chemotherapy: new era of 1(st)-line treatment for non-small-cell lung cancer. Mol Ther Oncolytics. 2019;13:1-6. https://doi.org/10.1016/j.omto.2019.02.001 30976658 PMC6444295

[7] Cheng Y , Zhang T, Xu Q. Therapeutic advances in non-small cell lung cancer: focus on clinical development of targeted therapy and immunotherapy. MedComm. 2021;2:692-729. https://doi.org/10.1002/mco2.105 34977873 PMC8706764

[8] Paz-Ares L , Luft A, Vicente D, et al; KEYNOTE-407 Investigators. Pembrolizumab plus chemotherapy for squamous non-small-cell lung cancer. N Engl J Med. 2018;379:2040-2051. https://doi.org/10.1056/NEJMoa1810865 30280635

[9] Gandhi L , Rodriguez-Abreu D, Gadgeel S, et al; KEYNOTE-189 Investigators. Pembrolizumab plus chemotherapy in metastatic non-small-cell lung cancer. N Engl J Med. 2018;378:2078-2092. https://doi.org/10.1056/NEJMoa1801005 29658856

[10] Gadgeel S , Rodriguez-Abreu D, Speranza G, et al Updated analysis from KEYNOTE-189: pembrolizumab or placebo plus pemetrexed and platinum for previously untreated metastatic nonsquamous non-small-cell lung cancer. J Clin Oncol. 2020;38:1505-1517. https://doi.org/10.1200/JCO.19.03136 32150489

[11] Garassino MC , Gadgeel SM, Speranza G, et al 973MO KEYNOTE-189 5-year update: first-line pembrolizumab (pembro) + pemetrexed (pem) and platinum vs placebo (pbo) + pem and platinum for metastatic nonsquamous NSCLC. Ann Oncol. 2022;33:S992-S993. https://doi.org/10.1016/j.annonc.2022.07.1101

[12] Aggarwal H , Bayo K, Han Y, et al Real-world maintenance therapy and survival outcomes for pembrolizumab plus pemetrexed and platinum for non-small-cell lung cancer in USA. Immunotherapy. 2023;15:267-281. https://doi.org/10.2217/imt-2022-0166 36789638

[13] Ma X , Long L, Moon S, Adamson BJS, Baxi SS. Comparison of population characteristics in real-world clinical oncology databases in the US: Flatiron Health, SEER, and NPCR. medRxiv. Preprint Posted online June 7, 2023. https://doi.org/10.1101/2020.03.16.20037143

[14] Birnbaum B , Nussbaum N, Seidl-Rathkopf K, et al Model-assisted cohort selection with bias analysis for generating large-scale cohorts from the EHR for oncology research. arXiv. Preprint Posted online January 13, 2020. https://arxiv.org/abs/2001.09765

[15] Waterhouse D , Lam J, Betts KA, et al Real-world outcomes of immunotherapy-based regimens in first-line advanced non-small cell lung cancer. Lung Cancer. 2021;156:41-49. https://doi.org/10.1016/j.lungcan.2021.04.007 33894493

[16] Velcheti V , Hu X, Piperdi B, Burke T. Real-world outcomes of first-line pembrolizumab plus pemetrexed-carboplatin for metastatic nonsquamous NSCLC at US oncology practices. Sci Rep. 2021;11:9222. https://doi.org/10.1038/s41598-021-88453-8 33911121 PMC8080779

[17] Mytelka DS , Li L, Benoit K. Post-diagnosis weight loss as a prognostic factor in non-small cell lung cancer. J Cachexia Sarcopenia Muscle. 2018;9:86-92. https://doi.org/10.1002/jcsm.12253 29205930 PMC5803614

[18] Pinto JA , Vallejos CS, Raez LE, et al Gender and outcomes in non-small cell lung cancer: an old prognostic variable comes back for targeted therapy and immunotherapy? ESMO Open. 2018;3:e000344. https://doi.org/10.1136/esmoopen-2018-000344 29682332 PMC5905840

[19] Brody R , Zhang Y, Ballas M, et al PD-L1 expression in advanced NSCLC: insights into risk stratification and treatment selection from a systematic literature review. Lung Cancer. 2017;112:200-215. https://doi.org/10.1016/j.lungcan.2017.08.005 29191596

[20] Griffith SD , Miksad RA, Calkins G, et al Characterizing the feasibility and performance of real-world tumor progression end points and their association with overall survival in a large advanced non-small-cell lung cancer data set. JCO Clin Cancer Inform. 2019;3:1-13. https://doi.org/10.1200/CCI.19.00013 PMC6873982 31403818

[21] Chauvin JM , Zarour HM. TIGIT in cancer immunotherapy. J ImmunoTher Cancer. 2020;8:e000957. https://doi.org/10.1136/jitc-2020-000957 32900861 PMC7477968

[22] Herbst RS , Arkenau HT, Bendell J, et al Phase 1 expansion cohort of ramucirumab plus pembrolizumab in advanced treatment-naive NSCLC. J Thorac Oncol. 2021;16:289-298. https://doi.org/10.1016/j.jtho.2020.10.004 33068794

[23] Curigliano G , Gelderblom H, Mach N, et al Phase I/Ib clinical trial of sabatolimab, an anti-TIM-3 antibody, alone and in combination with spartalizumab, an anti-PD-1 antibody, in advanced solid tumors. Clin Cancer Res. 2021;27:3620-3629. https://doi.org/10.1158/1078-0432.CCR-20-4746 33883177

[24] Falchook GS , Ribas A, Davar D, et al Phase 1 trial of TIM-3 inhibitor cobolimab monotherapy and in combination with PD-1 inhibitors nivolumab or dostarlimab (AMBER). J Clin Oncol. 2022;40:2504. https://doi.org/10.1200/jco.2022.40.16_suppl.2504

[25] Rodriguez-Abreu D , Johnson ML, Hussein MA, et al Primary analysis of a randomized, double-blind, phase II study of the anti-TIGIT antibody tiragolumab (tira) plus atezolizumab (atezo) versus placebo plus atezo as first-line (1L) treatment in patients with PD-L1-selected NSCLC (CITYSCAPE). J Clin Oncol. 2020;38:9503. https://doi.org/10.1200/jco.2020.38.15_suppl.9503

[26] Jiao S , Xia W, Yamaguchi H, et al PARP inhibitor upregulates PD-L1 expression and enhances cancer-associated immunosuppression. Clin Cancer Res. 2017;23:3711-3720. https://doi.org/10.1158/1078-0432.CCR-16-3215 28167507 PMC5511572

[27] Ramalingam SS , Thara E, Awad MM, et al JASPER: Phase 2 trial of first-line niraparib plus pembrolizumab in patients with advanced non-small cell lung cancer. Cancer. 2022;128:65-74. https://doi.org/10.1002/cncr.33885 34478166 PMC9293160

[28] Ramalingam S , Arora S, Whipple Neibauer M, et al P83.02 Niraparib + pembrolizumab (pembro) versus placebo + pembro 1L maintenance therapy in advanced NSCLC: ZEAL-1L phase III study. J Thorac Oncol. 2021;16:S653-S654. https://doi.org/10.1016/j.jtho.2021.01.1197

[29] Ahn MJ , Bondarenko I, Kalinka E, et al Durvalumab in combination with olaparib versus durvalumab alone as maintenance therapy in metastatic NSCLC: the phase 2 ORION study. J Thorac Oncol. 2023;18:1594-1606. https://doi.org/10.1016/j.jtho.2023.06.013 37390980

[30] Gray JE , Owonikoko TK, Kato T, et al Randomized phase III study of first-line pembrolizumab plus pemetrexed/platinum followed by pembrolizumab and maintenance olaparib versus pemetrexed in patients with metastatic nonsquamous non-small cell lung cancer (NSCLC): KEYLYNK-006. J Clin Oncol. 2020;38:TPS9632. https://doi.org/10.1200/jco.2020.38.15_suppl.tps9632

[31] Gray JE , Owonikoko TK, Kato T, et al 1418TiP Randomized, placebo-controlled phase III study of 1L pembrolizumab (Pembro) plus carboplatin/taxane followed by pembro with or without maintenance olaparib in patients (Pts) with metastatic squamous non-small cell lung cancer (sqNSCLC): KEYLYNK-008. Ann Oncol. 2020;31:S896. https://doi.org/10.1016/j.annonc.2020.08.1732

[32] Merck announces KEYLYNK-008 trial evaluating KEYTRUDA^®^ (pembrolizumab) plus LYNPARZA^®^ (olaparib) for patients with metastatic squamous non-small cell lung cancer to stop for futility. News release. December 7, 2023. Accessed January 02, 2024. https://www.businesswire.com/news/home/20231207532705/en/Merck-Announces-KEYLYNK-008-Trial-Evaluating-KEYTRUDA%C2%AE-pembrolizumab-Plus-LYNPARZA%C2%AE-olaparib-for-Patients-With-Metastatic-Squamous-Non-Small-Cell-Lung-Cancer-to-Stop-for-Futility

