# Supplementary material for: Predictors of outcomes in advanced non-small cell lung cancer treated with pembrolizumab maintenance
Source: Oncologist. 2025 Aug 25;30(8):oyaf070. doi: 10.1093/oncolo/oyaf070 (PMC12376147; doi:10.1093/oncolo/oyaf070)
Supplement: oyaf070_suppl_Supplementary_Tables_S1-S7_Figures_S1-S4 [file oyaf070_suppl_supplementary_tables_s1-s7_figures_s1-s4.pdf]

Supplemental material

Supplemental material has been provided by the authors to give readers additional information about their work.

Supplement to: Predictors of outcomes in advanced non-small cell lung cancer treated with pembrolizumab maintenance

Supplemental methods……..…………………………………………………………………..…2

Supplemental results…………………………………………………………….……..………4

Figure S1. Treatment in the subgroup of patients with nonsquamous histology…………………………...4

Figure S2. Treatment in the subgroup of patients with squamous histology……………………………….7

Figure S3. rwTTNTD and rwOS by pemetrexed use in the 1L setting in patients with nonsquamous histology………………………………………………………..……………………………………10

Figure S4. rwTTNTD and rwOS by stage at diagnosis……………………….…………………..12

Table S1. Candidate risk factors…………………………………………………………………..14

Table S2. Associations between predictors and rwTTNTD in patients with squamous histology in multivariable analyses……………………………………………………………………………...24

Table S3. Associations between predictors and rwOS in patients with squamous histology in multivariable analyses ………………………………………………………………………………………26

Table S4. Associations between predictors and rwTTNTD in patients with nonsquamous histology in multivariable analyses……………………………………………………………………………...28

Table S5. Associations between predictors and rwOS in patients with nonsquamous histology in multivariable analyses……………………………………………………………………………...31

Table S6. Harrell's C-statistic for each machine-learning model for training and testing datasets….…...34

Table S7. Top 10 predictors for each approach……………………………..……………………..35

Supplemental methods

Sensitivity analysis using machine-learning models

A sensitivity analysis using machine-learning approaches was introduced following the observation of limitations associated with the univariable and multivariable analysis approaches to supplement the primary analyses for real-world overall survival (rwOS) outcomes. Multiple continuous risk factors and laboratory value predictors were included in the primary planned analyses. Continuous predictors were categorized based on clinically important reference points (eg, low, normal, and high laboratory values) to avoid a violation of Cox proportional hazard regression's linearity assumption; however, this approach models the upper and lower values within a category as having an equal risk of the outcome, thereby reducing the precision of analyses and potentially masking important trends. Because of the nature of electronic health records (EHR) data, a large portion of values was missing for many risk factors. Missing data cannot be directly modeled using traditional regression approaches; therefore, missing values were defined as a distinct category for each risk factor. Categorizing all risk factors resulted in small cell counts that were not conducive to regression methodology, and the optimal approach to combining categories was not obvious. Using the a priori rule to include predictors with a log-rank test $P \leq .1$ resulted in the inclusion of multiple correlated predictors in the model, inducing collinearity. Notably, the same patients tended to have missing data across multiple variables (eg, certain patients were less likely to have laboratory data), which induced collinearity. This was remedied by subjectively selecting the final variable list (eg, based on prioritizing variables of clinical importance or interest).

A machine-learning sensitivity analysis was conducted to assess the consistency of results while addressing these concerns. Three machine-learning models were fitted to assess predictors of rwOS: an elastic net model, a survival classification and regression tree (CART) model, and a survival random forest model. These approaches were selected to assess the impact of limitations of the Cox proportional hazard regression strategy, provide insight into the robustness of the original modeling

results, and demonstrate the extent to which machine-learning methods provide value when predicting time-to-event outcomes with censoring. All machine-learning models were fitted using R version 4.1.1 (R Foundation for Statistical Computing, Vienna, Austria).

The analytic dataset was split into separate training and testing datasets to perform machine-learning analyses conditional on sample size. Tuning parameters were selected using the training dataset based on optimizing model performance assessed using 10-fold cross validation (for elastic net and CART) or out-of-bag error (for random forest). Model performance was assessed on the testing dataset and quantified using a C-statistic that accounts for censoring (eg, metric quantifying model discrimination). As a control, a model was trained and tested using Cox proportional hazard using the same specification as the primary analysis. For each model, the top 10 most influential predictors were identified based on descending variable importance statistics. The top predictors across models were also identified.

Supplemental results

Figure S1. Treatment in the subgroup of patients with nonsquamous histology

A

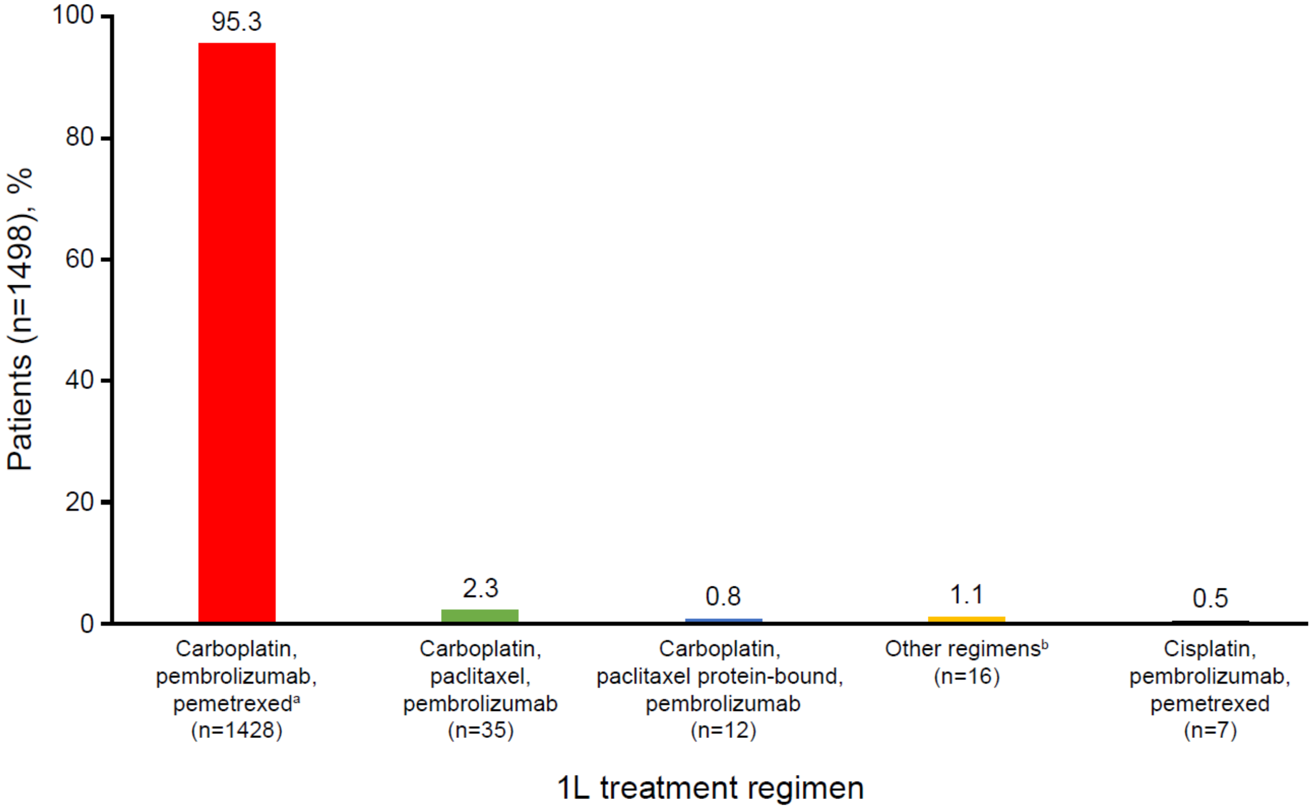

B

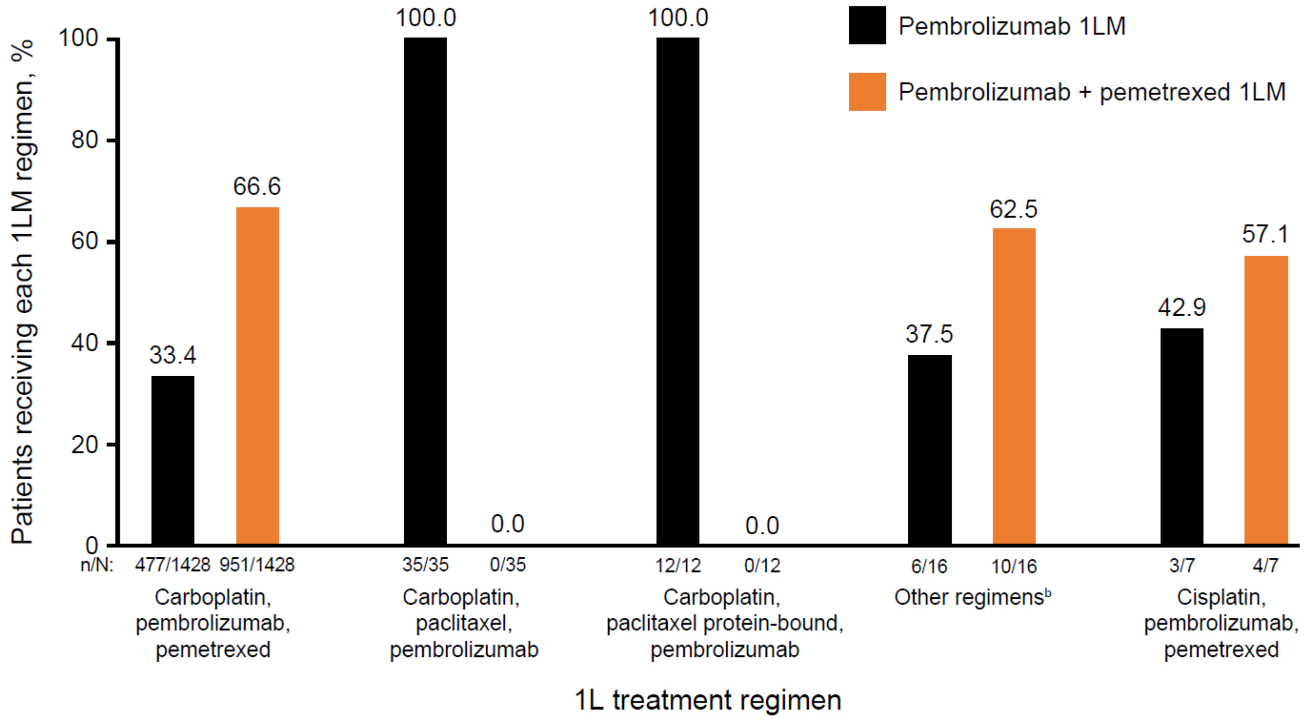
Pembrolizumab 1LM
Pembrolizumab + pemetrexed 1LM
Patients receiving each 1LM regimen, %
100
80
60
40
20
0
100.0
100.0
66.6
33.4
0.0
0.0
37.5
62.5
42.9
57.1
n/N:
477/1428
951/1428
35/35
0/35
12/12
0/12
6/16
10/16
3/7
4/7
Carboplatin, pembrolizumab, pemetrexed
Carboplatin, paclitaxel, pembrolizumab
Carboplatin, paclitaxel protein-bound, pembrolizumab
Other regimens[b]
Cisplatin, pembrolizumab, pemetrexed
1L treatment regimen

C

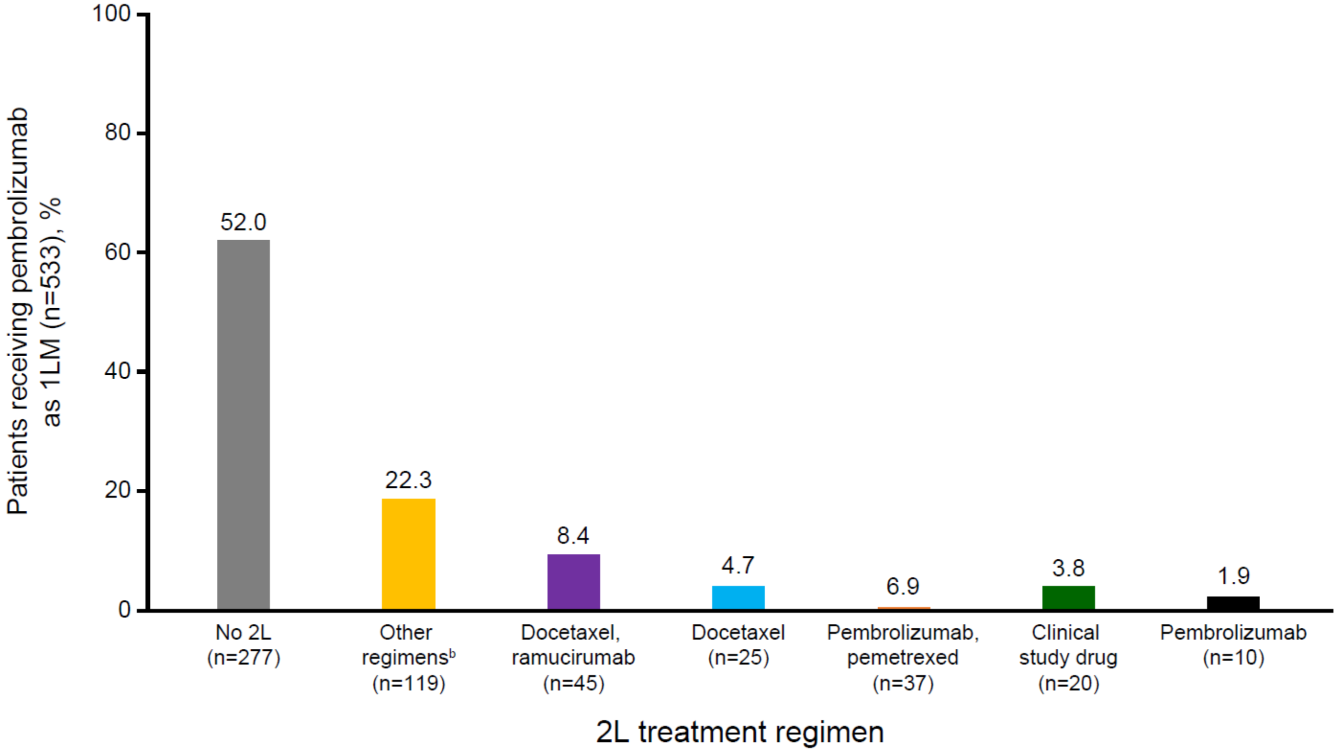
Patients receiving pembrolizumab as 1LM (n=533), %
100
80
60
40
20
0
52.0
22.3
8.4
4.7
6.9
3.8
1.9
No 2L (n=277)
Other regimens[b] (n=119)
Docetaxel, ramucirumab (n=45)
Docetaxel (n=25)
Pembrolizumab, pemetrexed (n=37)
Clinical study drug (n=20)
Pembrolizumab (n=10)
2L treatment regimen

D

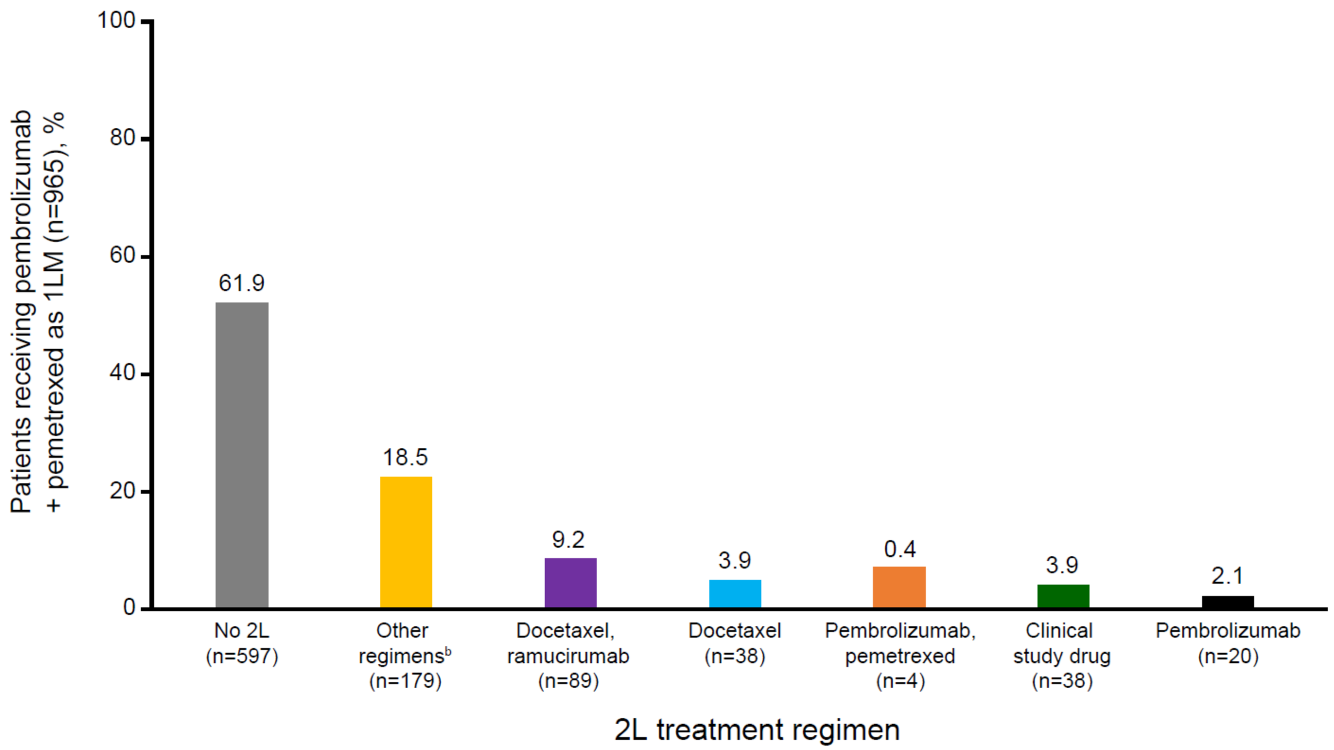

1L treatment regimen for patients with nonsquamous NSCLC (n = 1498) (**A**), proportion of patients with nonsquamous NSCLC who received pembrolizumab or pembrolizumab + pemetrexed as 1LM based on 1L treatment regimen (**B**), 2L treatment regimen for patients with nonsquamous NSCLC who received pembrolizumab as 1LM (n = 533) (**C**), and 2L treatment regimen for patients with nonsquamous NSCLC who received pembrolizumab + pemetrexed as 1LM (n = 965) (**D**).

Abbreviations: 1L, first-line; 1LM, first-line maintenance; 2L, second-line.

[a]This group includes 22 patients (1.5%) treated with abiraterone, carboplatin, pembrolizumab, and pemetrexed.

[b]Other regimens include regimens not defined in previous drug classes, such as sotorasib, capmatinib, and gemcitabine/vinorelbine.

Figure S2. Treatment in the subgroup of patients with squamous histology

A

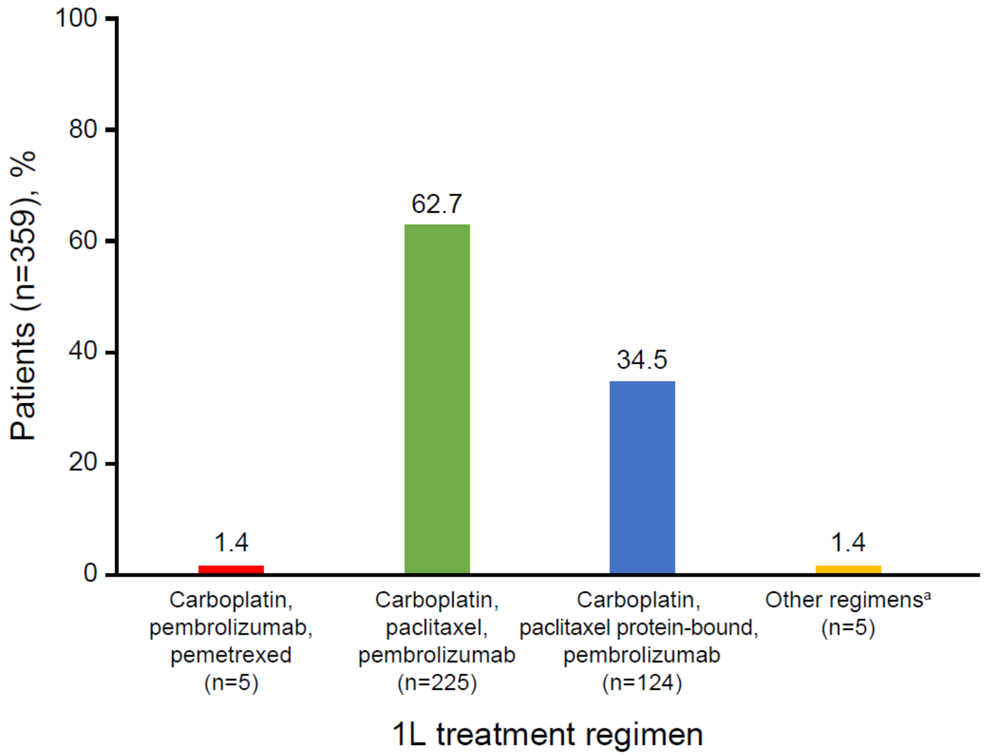

B

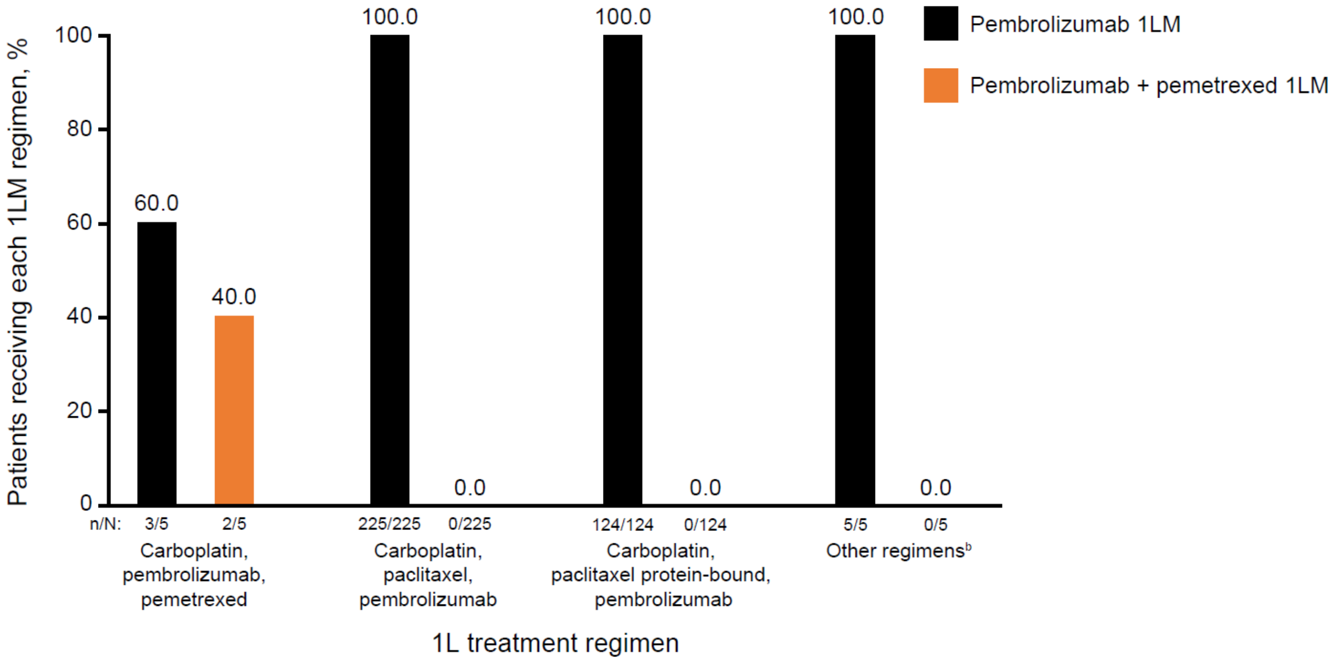
Patients receiving each 1LM regimen, %
Pembrolizumab 1LM
Pembrolizumab + pemetrexed 1LM
60.0
40.0
100.0
0.0
100.0
0.0
100.0
0.0
n/N: 3/5 2/5 225/225 0/225 124/124 0/124 5/5 0/5
Carboplatin, pembrolizumab, pemetrexed
Carboplatin, paclitaxel, pembrolizumab
Carboplatin, paclitaxel protein-bound, pembrolizumab
Other regimens[b]
1L treatment regimen

C

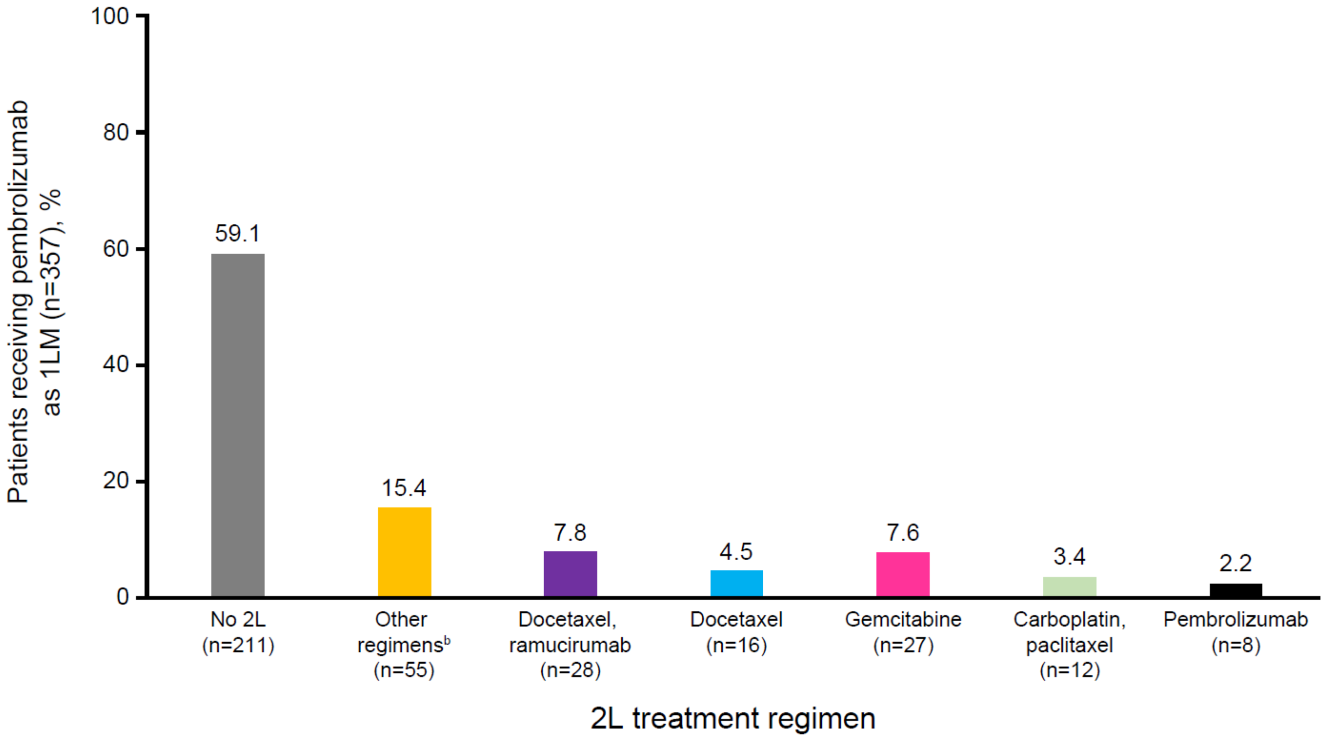
Patients receiving pembrolizumab as 1LM (n=357), %
59.1
15.4
7.8
4.5
7.6
3.4
2.2
No 2L (n=211)
Other regimens[b] (n=55)
Docetaxel, ramucirumab (n=28)
Docetaxel (n=16)
Gemcitabine (n=27)
Carboplatin, paclitaxel (n=12)
Pembrolizumab (n=8)
2L treatment regimen

D

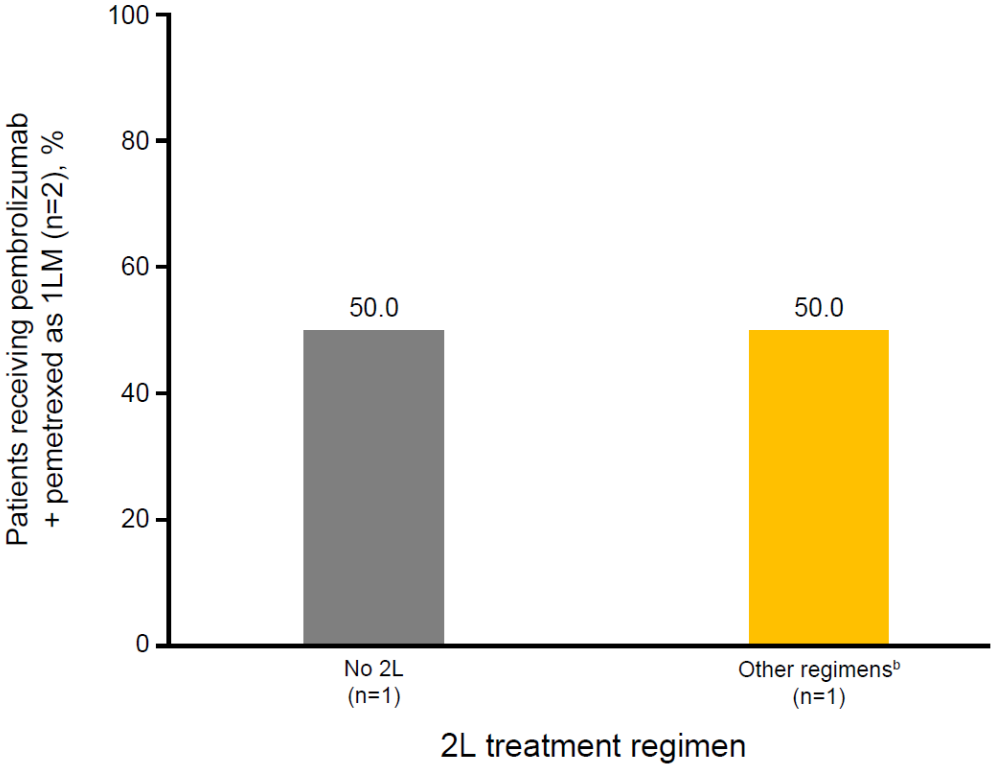

1L treatment regimen for patients with squamous NSCLC in the overall cohort (n = 359) (**A**), proportion of patients who received pembrolizumab or pembrolizumab + pemetrexed as 1LM based on 1L treatment regimen (**B**), 2L treatment regimen for patients with squamous NSCLC who received pembrolizumab as 1LM (n = 357) (**C**), and 2L treatment regimen for patients with squamous NSCLC who received pembrolizumab + pemetrexed as 1LM (n = 2) (**D**).

Abbreviations: 1L, first-line; 1LM, first-line maintenance; 2L, second-line.

[a]Other regimens include regimens not defined in previous drug classes, such as sotorasib, capmatinib, and gemcitabine/vinorelbine, as well as 1 patient with squamous histology treated with carboplatin, docetaxel, paclitaxel protein-bound, and pembrolizumab, and 1 patient with squamous histology treated with carboplatin, docetaxel, and pembrolizumab.

[b]Other regimens include regimens not defined in previous drug classes, such as sotorasib, capmatinib, and gemcitabine/vinorelbine.

Figure S3. (A) rwTTNTD and (B) rwOS by pemetrexed use in the 1L setting in patients with nonsquamous histology

A

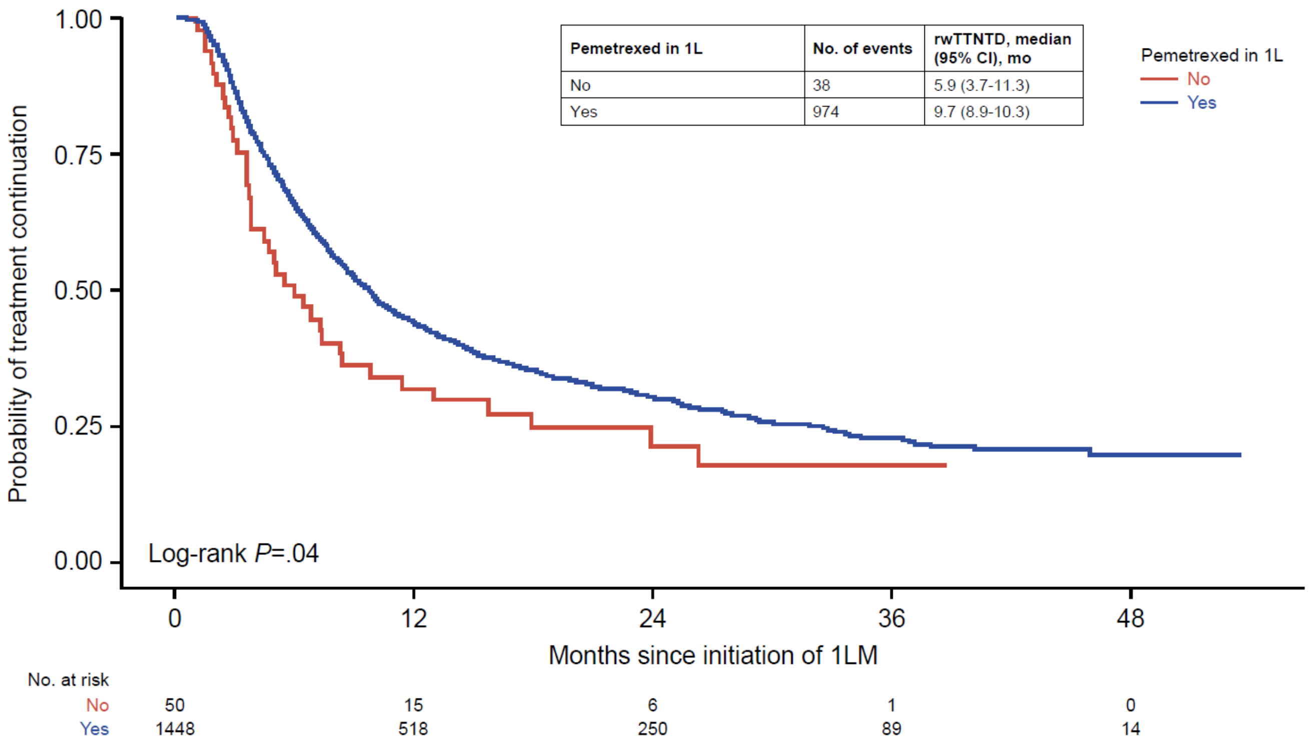

B

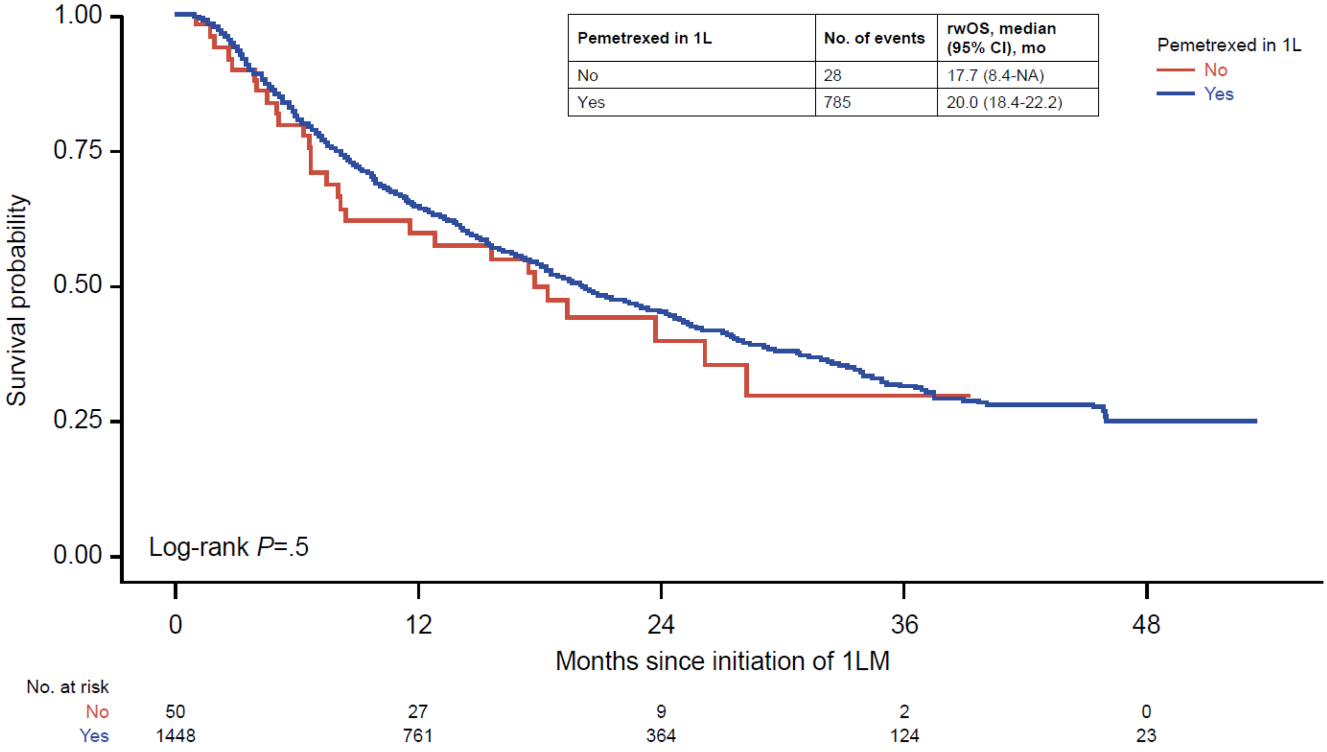

Abbreviations: 1L, first-line; 1LM, first-line maintenance; rwOS, real-world overall survival; rwTTNTD, real-world time to next treatment or death.

Figure S4. (A) rwTTNTD and (B) rwOS by stage at diagnosis

A

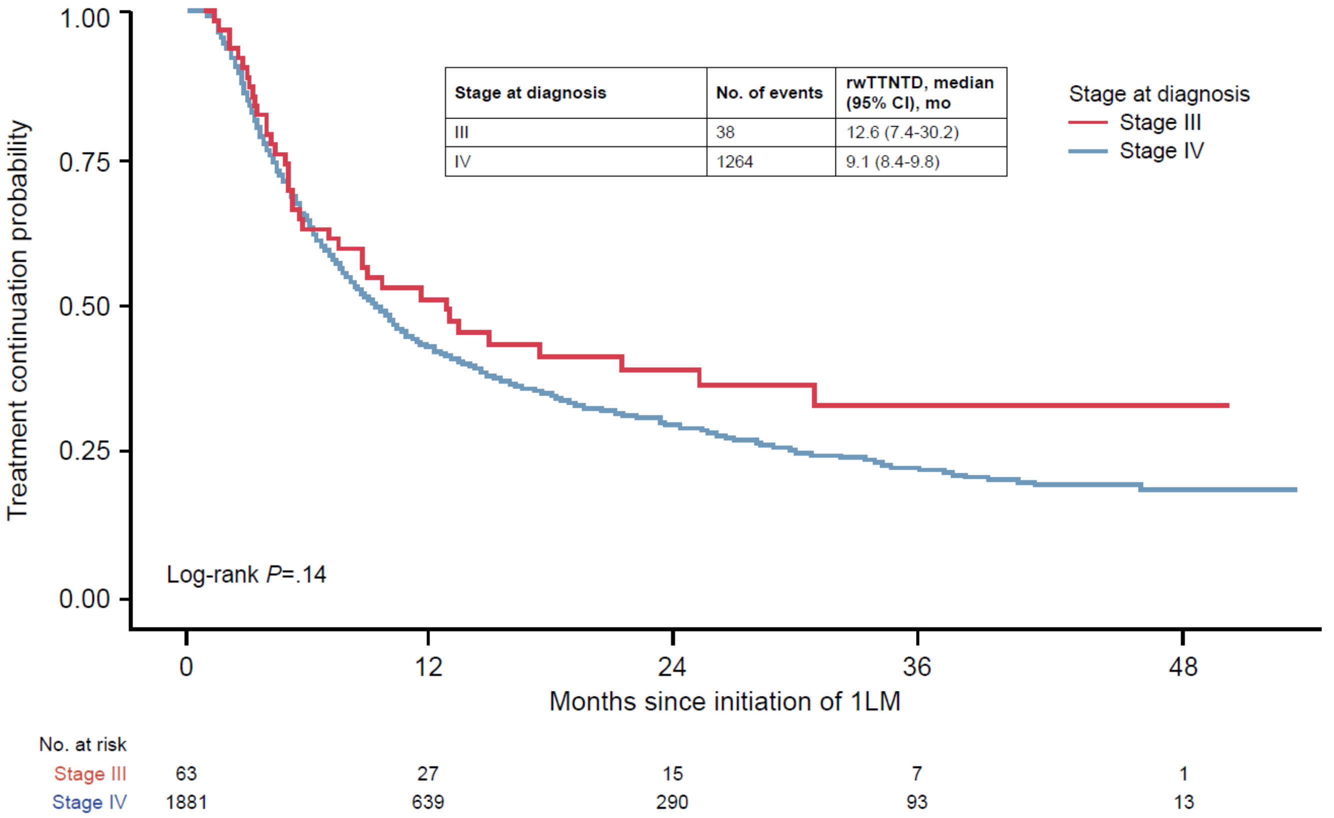

B

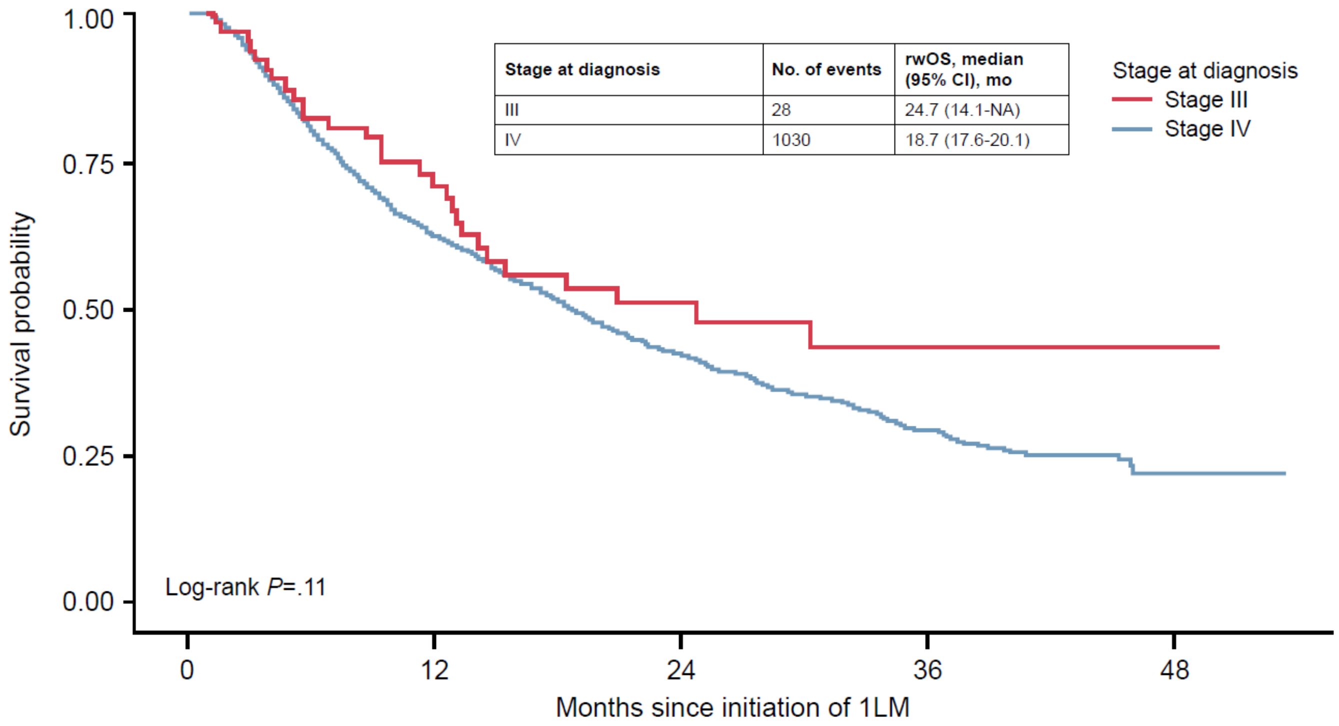

No. at risk					
Stage III	63	34	17	7	1
Stage IV	1881	941	416	130	22

Abbreviations: 1LM, first-line maintenance; rwOS, real-world overall survival; rwTTNTD, real-world time to next treatment or death.

Table S1. Candidate risk factors

**Demographic characteristics**	
Index year	Categories<br>• 2017<br>• 2018<br>• 2019<br>• 2020<br>• 2021
Age	Categories<br>• <60 years<br>• 60-69 years<br>• 70-79 years<br>• ≥80 years
Sex	Categories<br>• Male<br>• Female
BMI	Categories<br>• Underweight, BMI <18.5 kg/m^2<br>• Normal, 18.5-<25 kg/m^2<br>• Overweight, 25-<30 kg/m^2<br>• Obesity, ≥30 kg/m^2<br>• Unknown/not documented
Weight loss	Categories<br>• <5%, including no change/no weight loss<br>• 5%-<10%<br>• ≥10%

	• Unknown (no record to derive the variable)
Ethnicity	Categories<br>• Hispanic or Latino<br>• Not Hispanic or Latino<br>• Unknown/missing
Race	Categories<br>• Asian<br>• Black or African American<br>• White<br>• Other race<br>• Unknown/not documented
Smoking	Categories<br>• History of smoking<br>• No history of smoking<br>• Unknown/not documented
Practice type	Categories<br>• Community only<br>• Academic only<br>• Both community and academic<br>• Unknown/not documented
ECOG performance status	Categories<br>• 0-1<br>• >1<br>• Unknown/missing
Region of residence	Categories<br>• Northeast (states: CT, MA, ME, NH, RI, VT, NJ, NY, PA)

	• Midwest (states: IL, IN, MI, OH, WI, IA, KS, MN, MO, ND, NE, SD)<br>• South (states: DC, DE, FL, GA, MD, NC, SC, VA, WV, AL, KY, MS, TN, AR, LA, OK, TX)<br>• West (states: AZ, CO, ID, MT, NM, NV, UT, WY, AK, CA, HI, OR, WA)<br>• Other/unknown (PR) as defined at the index date
**Tumor clinicopathological characteristics**	
AJCC disease stage	Categories<br>• Stage IIIB<br>• Stage IIIC<br>• Stage IV
Histology	Categories<br>• Non–squamous cell carcinoma<br>• Squamous cell carcinoma<br>• NOS
Metastatic site	Categories<br>• Bone only (ICD-9: 198.5 secondary malignant neoplasm of bone and bone marrow; ICD-10: C79.51 secondary malignant neoplasm of bone)<br>• Liver only (ICD-9: 197.7 malignant neoplasm of liver, secondary; ICD-10: C78.7 secondary malignant neoplasm of liver and intrahepatic bile duct)<br>• Brain only (ICD-9: 198.3 for secondary

	malignant neoplasm of brain and spinal cord, and ICD-10: C79.31 for secondary malignant neoplasm of brain)<br>• Multiple sites with brain<br>• Multiple sites without brain<br>• Other sites/unknown sites (stage IV at initial diagnosis, but no record of metastasis site or site other than bone, liver, or brain)<br>• No metastasis
PD-L1	Categories<br>• PD-L1 staining <1% or 0%<br>• PD-L1 staining 1%-49%<br>• PD-L1 staining ≥50%<br>• Unknown (no interpretation given in report, results pending, unsuccessful/indeterminate test, unknown, not tested)
**Comorbidities**	
Myocardial infarction	Categories<br>• Yes<br>• No
Congestive heart failure	Categories<br>• Yes<br>• No
Peripheral vascular disease	Categories<br>• Yes<br>• No
Cerebrovascular disease	Categories

	• Yes<br>• No
Dementia	Categories<br>• Yes<br>• No
Interstitial lung disease	Categories<br>• Yes<br>• No
Chronic pulmonary disease	Categories<br>• Yes<br>• No
Rheumatologic disease	Categories<br>• Yes<br>• No
Peptic ulcer disease	Categories<br>• Yes<br>• No
Mild liver disease	Categories<br>• Yes<br>• No
Moderate or severe liver disease	Categories<br>• Yes<br>• No
Diabetes without chronic complications	Categories<br>• Yes<br>• No
Diabetes with chronic complications	Categories

	• Yes<br>• No
Hemiplegia or paraplegia	Categories<br>• Yes<br>• No
Renal disease	Categories<br>• Yes<br>• No
AIDS/HIV	Categories<br>• Yes<br>• No
**Laboratory measures**	
Hemoglobin	Categories<br>• Normal, if the level was within 13.5-17.5 g/dL in men or 12.0-15.5 g/dL in women<br>• Low, if the level was lower than the normal range<br>• High, if the level was higher than the normal range<br>• Unknown (no data available)
Aspartate aminotransferase	Categories<br>• Normal, if the level was within 8-33 U/L<br>• Low, if the level was lower than the normal range<br>• High, if the level was higher than the normal range<br>• Unknown (no data available)

Albumin	Categories<br>• Normal, if the level was within 34-54 g/L<br>• Low, if the level was lower than the normal range<br>• High, if the level was higher than the normal range<br>• Unknown (no data available)
Calcium	Categories<br>• Normal, if the level was within 8.6-10.3 mg/dL<br>• Low, if the level was lower than the normal range<br>• High, if the level was higher than the normal range<br>• Unknown (no data available)
Bilirubin	Categories<br>• Normal, if the level was within 0.2-1.2 mg/dL<br>• Low, if the level was lower than the normal range<br>• High, if the level was higher than the normal range<br>• Unknown (no data available)
Lymphocytes	Categories<br>• Normal, if the level was within 0.95-3.07 × 10^9/L<br>• Low, if the level was lower than the normal range<br>• High, if the level was higher than the normal range

	• Unknown (no data available)
Monocytes	Categories<br>• Normal, if the level was within 0.26-0.90 × 10^9/L<br>• Low, if the level was lower than the normal range<br>• High, if the level was higher than the normal range<br>• Unknown (no data available)
Platelets	Categories<br>• Normal, if the level was within 150-450 × 10^9/L<br>• Low, if the level was lower than the normal range<br>• High, if the level was higher than the normal range<br>• Unknown (no data available)
Neutrophils	Categories<br>• Normal, if the level was within 1.56-6.45 × 10^9/L<br>• Low, if the level was lower than the normal range<br>• High, if the level was higher than the normal range<br>• Unknown (no data available)
ALT	Categories<br>• Normal, if the level was within 7-55 U/L<br>• Low, if the level was lower than the normal range<br>• High, if the level was higher than the normal

	range<br>• Unknown (no data available)
Creatinine	Categories<br>• Normal, if the level was within 0.74-1.35 mg/dL in men or 0.59-1.04 mg/dL in women<br>• Low, if the level was lower than the normal range<br>• High, if the level was higher than the normal range<br>• Unknown (no data available)
NLR	Categories<br>• Low, ≤median<br>• High, >median<br>• Unknown if the levels of platelets or lymphocytes were unknown
PLR	Categories<br>• Low, ≤median<br>• High, >median<br>• Unknown if the levels of platelets or lymphocytes were unknown
LIPI	Categories<br>• Good, 0 factor<br>• Intermediate, 1 factor<br>• Poor, 2 factors<br>• Unknown/not documented
**Induction treatment**	
1L therapy cycle	Categories

	• 4 cycles<br>• 5 cycles<br>• 6 cycles
Pemetrexed in 1L	Categories<br>• Yes<br>• No

Abbreviations: 1L, first-line; AJCC, The American Joint Committee on Cancer; ALT, alanine transaminase; BMI, body mass index; ECOG, Eastern Cooperative Oncology Group; ICD, *International Classification of Diseases*; LIPI, lung immune prognostic index; NLR, neutrophil-to-lymphocyte ratio; NOS, not otherwise specified; PD-L1, programmed death-ligand 1; PLR, platelet-to-lymphocyte ratio.

Table S2. Associations between predictors and rwTTNTD in patients with squamous histology in multivariable analyses

**Predictor**	**HR**	**95% CI**	***P***
Index year vs 2018			
2019	1.2	0.5-2.7	.7
2020	1.4	0.6-3.2	.5
2021	1.5	0.6-3.6	.3
Index age vs 18-64 years			
65-79 years	1.1	0.8-1.6	.4
≥80 years	1.1	0.7-1.8	.6
Sex vs male			
Female	0.8	0.6-1.1	.2
Region vs South/other			
Northeast	1.3	0.9-2.0	.2
Midwest	1.3	0.9-1.9	.2
West	1.1	0.7-1.8	.6
Unknown	0.6	0.3-1.2	.2
ECOG PS vs 0-1			
>2	1.2	0.8-1.8	.4
Unknown	1.4	0.9-2.1	.2
Albumin levels vs normal/unknown			
Abnormal	1.3	0.9-1.9	.2
Monocyte levels vs normal			
Low	0.5	0.3-0.9	.02
High	1.7	1.1-2.6	.01
Unknown	1.0	0.5-1.8	.9
Alanine aminotransferase levels vs normal/unknown			

Low	1.7	1.0-3.0	.05
High	0.8	0.3-2.2	.7
Creatinine levels vs normal/unknown			
Low	1.1	0.8-1.5	.6
High	1.2	0.7-2.1	.5
NLR vs low/≤median			
High/>median	1.0	0.7-1.3	.8
Unknown	1.4	0.9-2.1	.1
PLR vs low/≤median			
High/>median	1.3	1.0-1.9	.07
Unknown	1.5	0.8-2.5	.2

Abbreviations: ECOG PS, Eastern Cooperative Oncology Group performance status; HR, hazard ratio; NLR, neutrophil-to-lymphocyte ratio; PD-L1, programmed death-ligand 1; PLR, platelet-to-lymphocyte ratio; rwTTNTD, real-world time to next treatment or death.

Table S3. Associations between predictors and rwOS in patients with squamous histology in multivariable analyses

Predictor	HR	95% CI	*P*
Index year vs 2018			
2019	1.1	0.4-2.9	.8
2020	1.2	0.5-3.2	.7
2021	1.4	0.5-3.7	.5
Index age vs 18-64 years			
65-79 years	1.0	0.7-1.4	.9
≥80 years	1.1	0.6-1.8	.8
Sex vs male			
Female	0.7	0.5-1.0	.03
Region vs South/other			
Northeast	1.2	0.8-1.9	.3
Midwest	1.3	0.9-2.0	.2
West	0.8	0.5-1.4	.5
Unknown	0.3	0.2-0.7	<.001
Weight loss vs no weight loss, <5%, or missing			
5%-<10%	1.7	1.1-2.4	.01
≥10%	1.6	1.0-2.5	.07
ECOG PS vs 0-1			
>2	1.1	0.7-1.7	.6
Unknown	1.2	0.7-1.9	.5
PD-L1 status vs 0%-<1%			
1%-49%	1.0	0.7-1.4	.9
≥50%	0.5	0.3-0.8	.01
Unknown	0.9	0.6-1.3	.5

Monocyte levels vs normal			
Low	0.5	0.3-1.0	.04
High	1.5	1.0-2.4	.07
Unknown	1.1	0.5-2.2	.8
Alanine aminotransferase levels vs normal/unknown			
Low	1.6	0.9-3.0	.1
High	1.9	0.7-5.1	.2
NLR vs low/≤median			
High/>median	1.0	0.7-1.5	1.0
Unknown	1.4	0.9-2.1	.2
PLR vs low/≤median			
High/>median	1.6	1.1-2.3	.01
Unknown	1.8	1.0-3.4	.05

Abbreviations: ECOG PS, Eastern Cooperative Oncology Group performance status; HR, hazard ratio; NLR, neutrophil-to-lymphocyte ratio; PD-L1, programmed death-ligand 1; PLR, platelet-to-lymphocyte ratio; rwOS, real-world overall survival.

Table S4. Associations between predictors and rwTTNTD in patients with nonsquamous histology in multivariable analyses

Predictor	HR	95% CI	*P*
Index year vs 2018			
2017	0.9	0.7-1.3	.7
2019	0.9	0.8-1.1	.3
2020	1.0	0.8-1.2	1.0
2021	1.1	0.9-1.3	.5
Index age vs 18-64 years			
65-79 years	0.9	0.8-1.1	.3
≥80 years	1.0	0.8-1.2	.9
Sex vs male			
Female	0.9	0.8-1.0	.03
Ethnicity vs not Hispanic/Latino			
Hispanic/Latino	1.2	0.8-1.7	.4
Unknown/missing	1.2	1.0-1.4	.01
Region vs South/other			
Northeast	1.1	1.0-1.4	.2
Midwest	1.0	0.8-1.2	.9
West	1.0	0.8-1.3	.7
Unknown	1.1	0.8-1.3	.7
Weight loss vs no weight loss, <5%, or missing			
5%-<10%	1.4	1.2-1.6	<.001
≥10%	1.8	1.4-2.1	<.001
Stage at index vs III			
IV	1.8	1.2-2.8	.01
ECOG PS vs 0-1			

>2	1.1	0.9-1.4	.2
Unknown	1.2	1.0-1.5	.03
Smoking history vs no history of smoking			
History of smoking	0.8	0.7-1.0	.1
PD-L1 status vs 0%-<1%			
1%-49%	0.8	0.7-0.9	.01
≥50%	0.5	0.4-0.6	<.001
Unknown	0.8	0.7-0.9	.01
Pemetrexed in 1L vs no			
Yes	0.7	0.5-1.0	.07
Hemoglobin levels vs normal/unknown			
Abnormal	1.3	1.0-1.6	.03
Albumin levels vs normal/unknown			
Abnormal	1.2	1.0-1.4	.1
Calcium levels vs normal/unknown			
Low	1.5	1.2-1.8	0
High	1.1	0.7-1.6	.8
Monocyte levels vs normal			
Low	0.9	0.7-1.2	.5
High	1.2	1.0-1.4	.03
Unknown	0.9	0.7-1.1	.4
Alanine aminotransferase levels vs normal/unknown			
Low	1.1	0.7-1.7	.6
High	0.9	0.7-1.1	.3
Creatinine levels vs normal/unknown			
Low	1.3	1.1-1.5	.01
High	1.0	0.8-1.2	.98

NLR vs low/≤median			
High/>median	1.2	1.0-1.4	.02
Unknown	1.2	1.0-1.5	.02

Abbreviations: 1L, first-line; ECOG PS, Eastern Cooperative Oncology Group performance status; HR, hazard ratio; NLR, neutrophil-to-lymphocyte ratio; PD-L1, programmed death-ligand 1; rwTTNTD, real-world time to next treatment or death.

Table S5. Associations between predictors and rwOS in patients with nonsquamous histology in multivariable analyses

**Predictor**	**HR**	**95% CI**	***P***
Index year vs 2018			
2017	0.7	0.5-1.0	.1
2019	1.0	0.8-1.2	.8
2020	1.0	0.8-1.3	.9
2021	1.2	0.9-1.6	.1
Index age vs 18-64 years			
65-79 years	0.9	0.8-1.1	.5
≥80 years	1.1	0.9-1.5	.3
Sex vs male			
Female	0.8	0.7-1.0	.02
Region vs South/other			
Northeast	1.1	0.9-1.3	.6
Midwest	1.3	1.0-1.6	.03
West	1.1	0.9-1.4	.4
Unknown	1.1	0.9-1.4	.5
Weight loss vs no weight loss, <5%, or missing			
5%-<10%	1.6	1.3-1.9	<.001
≥10%	2.1	1.7-2.6	<.001
Stage at index vs III			
IV	2.5	1.4-4.4	<.001
ECOG PS vs 0-1			
>2	1.4	1.1-1.7	<.001
Unknown	1.1	0.9-1.4	.2
PD-L1 status vs 0%-<1%			

1%-49%	0.8	0.7-1.0	.05
≥50%	0.6	0.5-0.7	<.001
Unknown	0.8	0.7-1.0	.1
Cardiovascular disease vs no			
Yes	1.1	0.8-1.4	.6
Pemetrexed in 1L vs no			
Yes	1.0	0.7-1.5	1.0
Hemoglobin levels vs normal/unknown			
Abnormal	1.4	1.1-1.8	.01
Albumin levels vs normal/unknown			
Abnormal	1.3	1.1-1.7	.01
Calcium levels vs normal/unknown			
Low	1.3	1.1-1.7	.01
High	0.9	0.5-1.5	.6
Monocyte levels vs normal			
Low	0.9	0.7-1.2	.4
High	1.2	1.0-1.4	.1
Unknown	0.9	0.7-1.2	.5
Alanine aminotransferase levels vs normal/unknown			
Low	1.4	0.9-2.2	.2
High	0.7	0.5-1.0	.05
Creatinine levels vs normal/unknown			
Low	1.4	1.1-1.7	<.001
High	1.1	0.8-1.4	.5
NLR vs low/≤median			
High/>median	1.2	1.0-1.4	.06
Unknown	1.4	1.1-1.7	<.001

PLR vs low/≤median			
High/>median	1.1	0.9-1.3	.2
Unknown	0.9	0.7-1.2	.6

Abbreviations: 1L, first-line; ECOG PS, Eastern Cooperative Oncology Group performance status; HR, hazard ratio; NLR, neutrophil-to-lymphocyte ratio; PD-L1, programmed death-ligand 1; PLR, platelet-to-lymphocyte ratio; rwOS, real-world overall survival.

Table S6. Harrell's C-statistic for each machine-learning model for the training and testing datasets

Model	C-index, training dataset	C-index, testing dataset
Model 1: Cox proportional hazards regression	0.65	0.64
Model 2a: Elastic net regression, requiring the inclusion of core predictors	0.64	0.63
Model 2b: Elastic net regression, full variable selection	0.64	0.66
Model 2c: Elastic net regression, full variable selection, continuous predictors	0.64	0.64
Model 3a: CART, without imputation	0.58	0.56
Model 3b: CART, with imputation	0.58	0.56
Model 4a: Random forest, without imputation	0.62	0.64
Model 4b: Random forest, continuous predictors with imputation	0.63	0.65

Abbreviation: CART, classification and regression tree.

Table S7. Top 10 predictors for each approach

Rank	Cox proportional hazards regression	Elastic net	CART	Random forest
1	Weight loss: ≥10% vs <5% or missing	Weight loss:[b] ≥10% vs <5% or missing	Weight loss: ≥10% vs <5% or missing	Weight loss
2	Weight loss: 5%-10% vs <5% or missing	Albumin: low vs high, normal, or unknown	–	Low albumin values
3	PD-L1 status:[a] ≥50% vs 0%-<1%	Weight loss: 5%-10% vs <5% or missing	–	Low hemoglobin values
4	Creatinine: low vs normal/unknown	Creatinine: low vs high, normal, or unknown	–	High bilirubin values
5	Sex:[a] female vs male	PD-L1 status:[a] ≥50% vs <50%	–	Low aspartate values
6	NLR: unknown vs low, ≤median	BMI: underweight vs normal, overweight, obese, or unknown	–	Low and high calcium values
7	PLR: high, >median vs low, ≤median	Sex:[a] female vs male	–	Low ALT values
8	PD-L1 status:[a] ≥1%-49% vs 0%-<1%	ALT: high vs normal or unknown	–	Low creatinine values
9	Region: Midwest vs South/other	Neutrophil: high vs normal or unknown	–	High monocyte values

10	Albumin: abnormal (low or high) vs normal/unknown	ALT: high vs normal or unknown	–	High platelet values

Abbreviations: ALT, alanine transaminase; BMI, body mass index; CART, classification and regression tree; NLR, neutrophil-to-lymphocyte ratio; PD-L1, programmed death-ligand 1; PLR, platelet-to-lymphocyte ratio; rwOS, real-world overall survival.

[a]Denotes protective factor for rwOS. All other predictors were identified as risk factors.

[b]During the period between the initial diagnosis and the index date.
